# OSD1 Promotes Meiotic Progression via APC/C Inhibition and Forms a Regulatory Network with TDM and CYCA1;2/TAM

**DOI:** 10.1371/journal.pgen.1002865

**Published:** 2012-07-26

**Authors:** Laurence Cromer, Jefri Heyman, Sandra Touati, Hirofumi Harashima, Emilie Araou, Chloe Girard, Christine Horlow, Katja Wassmann, Arp Schnittger, Lieven De Veylder, Raphael Mercier

**Affiliations:** 1 INRA, UMR1318, Institut Jean-Pierre Bourgin, Versailles, France; 2 AgroParisTech, Institut Jean-Pierre Bourgin, Versailles, France; 3 Department of Plant Systems Biology, VIB, Gent, Belgium; 4 Department of Plant Biotechnology and Bioinformatics, Ghent University, Gent, Belgium; 5 UMPC University of Paris 6, UMR7622, Paris, France; 6 CNRS, UMR7622, Laboratoire de Biologie du Développement, Paris, France; 7 IBMP, UPR2357 du CNRS, Strasbourg, France; 8 Trinationales Institut fuer Pflanzenforschung, Strasbourg, France; Cornell University, United States of America

## Abstract

Cell cycle control is modified at meiosis compared to mitosis, because two divisions follow a single DNA replication event. Cyclin-dependent kinases (CDKs) promote progression through both meiosis and mitosis, and a central regulator of their activity is the APC/C (Anaphase Promoting Complex/Cyclosome) that is especially required for exit from mitosis. We have shown previously that OSD1 is involved in entry into both meiosis I and meiosis II in *Arabidopsis thaliana*; however, the molecular mechanism by which OSD1 controls these transitions has remained unclear. Here we show that OSD1 promotes meiotic progression through APC/C inhibition. Next, we explored the functional relationships between OSD1 and the genes known to control meiotic cell cycle transitions in *Arabidopsis*. Like *osd1*, *cyca1;2/tam* mutation leads to a premature exit from meiosis after the first division, while *tdm* mutants perform an aberrant third meiotic division after normal meiosis I and II. Remarkably, while *tdm* is epistatic to *tam*, *osd1* is epistatic to *tdm*. We further show that the expression of a non-destructible *CYCA1;2/TAM* provokes, like *tdm*, the entry into a third meiotic division. Finally, we show that CYCA1;2/TAM forms an active complex with CDKA;1 that can phosphorylate OSD1 *in vitro*. We thus propose that a functional network composed of OSD1, CYCA1;2/TAM, and TDM controls three key steps of meiotic progression, in which OSD1 is a meiotic APC/C inhibitor.

## Author Summary

In the life cycle of sexual organisms, a specialized cell division—meiosis—reduces the number of chromosomes from two sets (2n, diploid) to one set (n, haploid), while fertilization restores the original chromosome number. Meiosis reduces ploidy because it consists of two cellular divisions following a single DNA replication. In this study, we analyze the function of a group of genes that collectively controls the entry into the first meiotic division, the entry into the second meiotic division, and the exit from meiosis in the model plant *Arabidopsis thaliana*. We revealed a complex regulation network that controls these three key transitions.

## Introduction

Meiosis is a key step in the life cycle of sexually reproducing eukaryotes, such as the majority of flowering plants. At meiosis ploidy is reduced by two, leading to the production of typically haploid gametes whose fusion during fertilization restores diploidy of the next generation. This is achieved by the modification of the meiotic cell cycle, compared to mitosis, allowing two rounds of chromosome segregation – meiosis I and meiosis II - after a single DNA replication event. Thus, a central question when analyzing the meiotic cell cycle is how three key transitions are controlled, i.e. entry of the meiocytes into meiosis I after prophase, transition from meiosis I to meiosis II and exit from meiosis II.

The main driving force of cell-cycle progression, at both meiosis and mitosis, is the activity of cyclin-dependent kinases (CDKs), in association with their regulatory partners, the cyclins. Entry into division phase requires high CDK activity that peaks at metaphase. Anaphase progression is regulated by a gradual degradation of CDK activity and mitotic exit requires low CDK activity [1], [2]. CDK activity is regulated by the anaphase-promoting complex/cyclosome (APC/C), a conserved multisubunit E3 ubiquitin ligase that triggers the degradation of multiple substrates, including cyclins, during mitosis and meiosis. The APC/C is activated by Cdc20/Fizzy and Cdh1/Fizzy-related proteins that also confer substrate specificity (the latter is known as CCS52s in plants) [3]–[5]. Precisely how the mitotic machinery is modified for the purpose of meiosis is unclear. The currently available knowledge that originates from studies carried out in unicellular fungi, *Xenopus laevis* and mouse oocyte systems, points towards a meiosis specific regulation of the APC/C as one of the key cell cycle modifications between meiosis and mitosis [2], [3]. In oocytes, meiosis is driven by Cdc2/Cyclin B complexes. At the end of meiosis I, Cyclin B is only partially degraded and the residual, low level of Cdc2/CyclinB activity is essential for entry into meiosis II [6]. Partial Cyclin B degradation is obtained through temporally controlled inhibition of the APC/C by the Erp1/Emi2 protein [7], [8]. In *Schizosaccharomyces pombe*, the Mes1 protein also partially restrains cyclin degradation through inhibition of the APC/C, thereby allowing entry into meiosis II [9]–[11]. In S*accharomyces cerevisiae*, a meiosis specific APC/C activator (Ama1), and its inhibitor Mnd2, are both required for meiotic progression [3].

Very little is known about control of the meiotic cell cycle in plants. It is largely unknown which one(s) of the *Arabidopsis* cyclins (which include 10 A-type-cyclins and 11 B-type-cyclins) constitute, with CDKA;1 [12]–[14] and possibly other CDKs, the core CDK complex that is necessary for meiosis. To date, only four genes involved in the three meiotic cell cycle transitions have been isolated in *Arabidopsis thaliana*. Two of them, *TAM* (*TARDY ASYNCHRONOUS MEIOSIS*, also known as *CYCA1;2*) and *OSD1* (*OMISSION OF SECOND DIVISION*) are essential for the meiosis I/meiosis II transition. The mutation of *CYCA1;2/TAM* or of *OSD1* leads to a premature exit from meiosis after meiosis I, and thus to the production of diploid spores and gametes [15]–[18]. These two genes are also involved in the prophase/meiosis I transition as their concomitant loss leads to a premature exit from meiosis after prophase I, before any division [15]. *CYCA1;2/TAM* encodes one of the 10 *Arabidopsis* A-type cyclins [18] and *OSD1* encodes a plant-specific protein, with additional functions in suppressing ectopic endomitosis via APC/C inhibition [15], [16], [19]. The third one, *TDM* (*THREE-DIVISION MUTANT*), is essential for meiotic exit, as its mutation leads to entry into an aberrant third division of meiosis after regular first and second divisions [13], [20], [21]. Finally, *SMG7* (*SUPPRESSOR WITH MORPHOGENETIC EFFECTS ON GENITALIA 7*) is also required at the end of meiosis, as its mutation leads to cell cycle arrest at anaphase II [13], [22]. Epistasis analysis suggest that SMG7 and TDM act in the same pathway [13].

Here we explored the meiotic molecular function of OSD1 and CYCA1;2/TAM, and the functional relationship between OSD1, the APC/C, CYCA1;2/TAM and TDM to control meiosis progression.

## Results

### OSD1 shares structural similarities with other APC/C inhibitors

**Figure 1:**
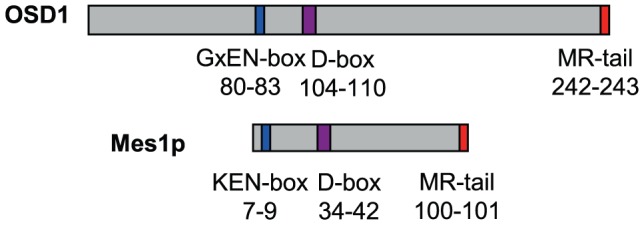
Structural comparison of OSD1 and Mes1 proteins. OSD1 and Mes1 share co-aligned putative APC/C interacting domains.

OSD1 depletion leads to a premature exit from meiosis at the end of meiosis I, a phenotype reminiscent of the vertebrate Erp1/Emi2-depleted oocytes and the *mes1* fission yeast mutant. While this work was in progress, evidence was found that OSD1 (also named GIGAS CELL 1, GIG1) negatively regulates the APC/C to control mitotic progression [19]. Yet, while the OSD1 protein has been shown to act as a mitotic APC/C inhibitor [19] and is well conserved in all plants, it does not appear to be conserved over other eukaryotes and notably does not show global similarity with other known APC/C inhibitors [16], which conversely do not seem to have homologues in plants. However, closer examination of the OSD1 sequence revealed that OSD1 shares multiple features with Mes1: OSD1 has the same three putative cell-cycle-related domains in the same order on the protein (Figure 1). These three domains are very well conserved over OSD1 homologues (Figure S1) [16]. Two of these domains are putative APC/C degradation motifs: a D-box (residues 104–110, RxxLxx[LIVM]) and a GxEN/KEN-box (residues 80–83, GxEN in eudicotyledon and KEN in monocotyledon OSD1 homologues). The corresponding two motifs have been shown to be important for the Mes1 function [10]. OSD1 also has a C-terminal MR-tail in common with Mes1 (the two last amino-acids of the protein are a methionine and an arginine). This MR-tail has not been functionally tested in Mes1. However the MR-tail of Nek2a, a kinase that is involved in mitotic regulation via APC/C inhibition, has been described as being a docking domain of Nek2a on the APC/C, being thus essential for its binding and inhibition activities [23]. Similarly, the C-terminal RL-tail of Emi2 is essential for inhibition of the APC/C at meiosis [24]. These observations prompted us to propose that OSD1 might also promote meiotic progression by regulating the APC/C activity through these three domains.

### OSD1 interacts with activator subunits of the APC/C via its conserved domains

**Figure 2:**
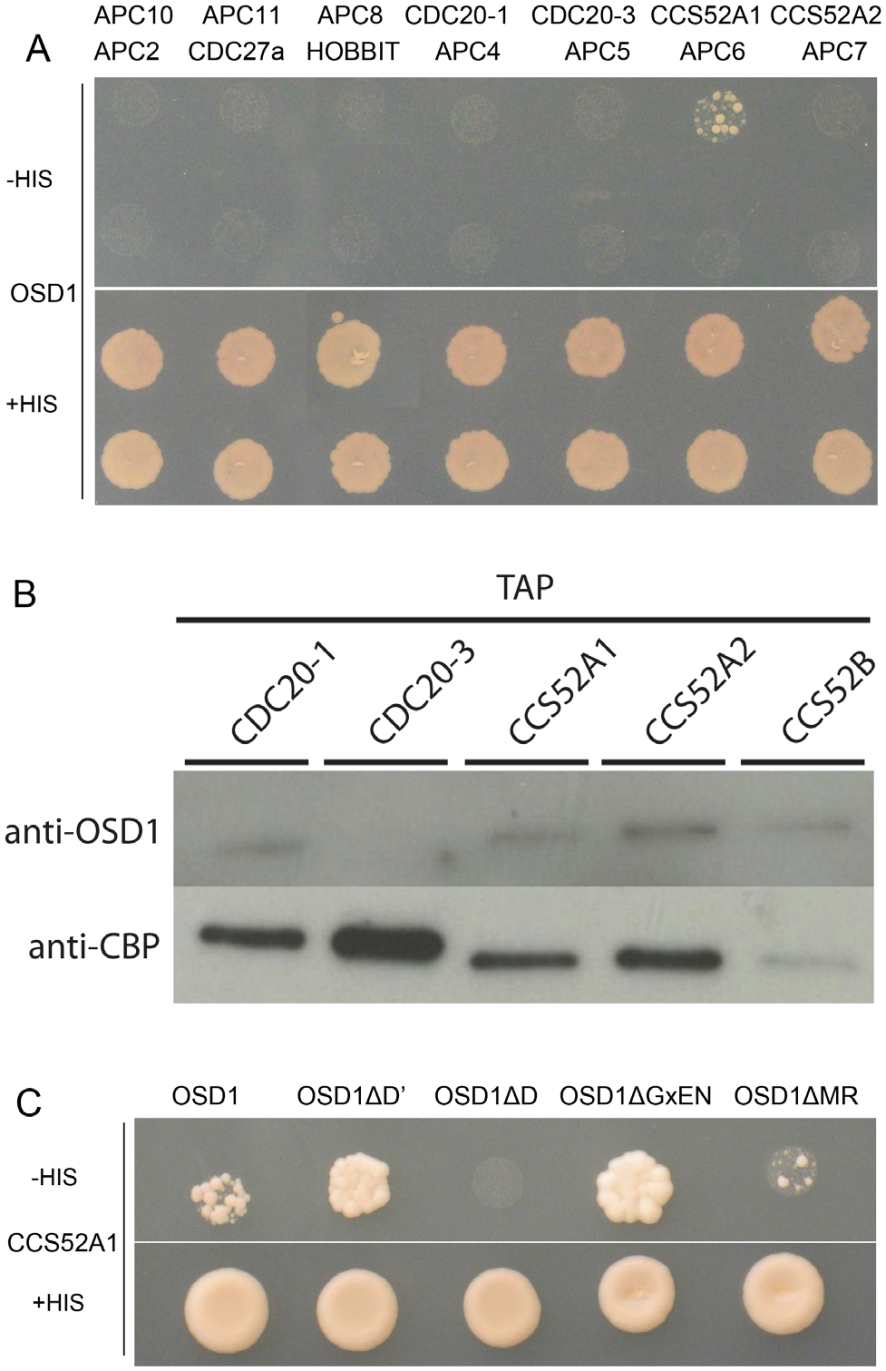
OSD1 interacts with CCS52A1 through its D-BOX and MR-tail. (A) Yeast 2-hybrid experiments showed that among the 14 APC/C subunits tested, OSD1 interacts only with CCS52A1. (B) CDC20s and CCS52s TAP elutions probed with anti-OSD1 antibody. OSD1 is detected in all elutions but CDC20.3. The anti-CBP recognizes the Calmodulin Binding Protein stretch in the TAP tag and served as loading control. (C) OSD1 with mutation of a putative non-conserved D-BOX (OSD1ΔD') or mutation of its GxEN-box (OSD1ΔGxEN) still interacts with CCS52A1. In contrast, this interaction is abolished by the mutation of the conserved D-box (OSD1ΔD) and reduced by the deletion of the MR-tail (OSD1ΔMR).

Using yeast 2-hybrid (Y2H) experiments Iwata et al [19] recently showed that OSD1 (also called GIG1) interacts with the APC/C activator CDC20.1, CDC20.5, CCS52A1 and CCS52B, but not with the core APC/C components they tested (APC2, APC7, APC10, CDC27a, and HBT). We independently used Y2H experiments to test interaction of OSD1 with different APC/C subunits (Figure 2A). Corroborating and extending Iwata et al results, OSD1 did not interact with any of the APC/C core subunits tested (APC2, CDC27a, HBT, APC4, APC5, APC6, APC7, APC8, APC10, APC11). Concerning the activators, our result confirmed the interaction with CCS52A1 but did not reveal interaction with the other activators tested, including CDC20.1 that was scored positively by Iwata et al. As a negative result in Y2H experiments could be due to protocol and material variations, we used a complementary approach. Tandem affinity purification (TAP) experiments, using APC/C core components and the activators CCS52A2, CCS52B and CDC20.1 as baits, previously identified OSD1 by mass spectrometry [25]. As mass spectrometry can fail to identify all proteins in a sample, we used an anti-OSD1 antibody (Figure S2) on TAP purified samples using CDC20.1, CDC20-3 and the three CDH1 homologues (CCS52A1, CCS52A2, CCS52B) as bait [25], to test to presence of OSD1. OSD1 was revealed in the CDC20.1 TAP (but not CDC20-3) and the three CCS52 TAPs (Figure 2B). Altogether our and Iwata *et al* results suggest that OSD1 can interact with a range of APC/C activators, including CDC20.1, CDC20.5, CCS52A1, CCS52A2 and CCS52B.

Next we asked whether the D-box, GxEN-box and MR tail represent true APC/C interaction motifs (Figure 2C). For both the D-box and GxEN-box, the amino acid residues essential for APC/C binding were substituted to alanine (ΔD, RxxL→AxxA; G, GxEN→AxAA) whereas the MR tail was deleted (ΔMR). We also mutated a putative second D-box motif (ΔD′, RxxL Aa 34–37) in OSD1 that is not conserved among the different plant proteins (Figure S1). All the OSD1 proteins were stably expressed in yeast (Figure S3). Mutation of the conserved D-box completely abolished the Y2H interaction with CCS52A1. Deletion of the MR tail diminished, but not abolished the interaction with the APC/C activator. In contrast, mutation of D′ or of the GxEN-box did not reduce the interaction with CCS52A1 (Figure 2C).

### OSD1 function *in planta* is dependent on its D-box and MR-tail

**Figure 3:**
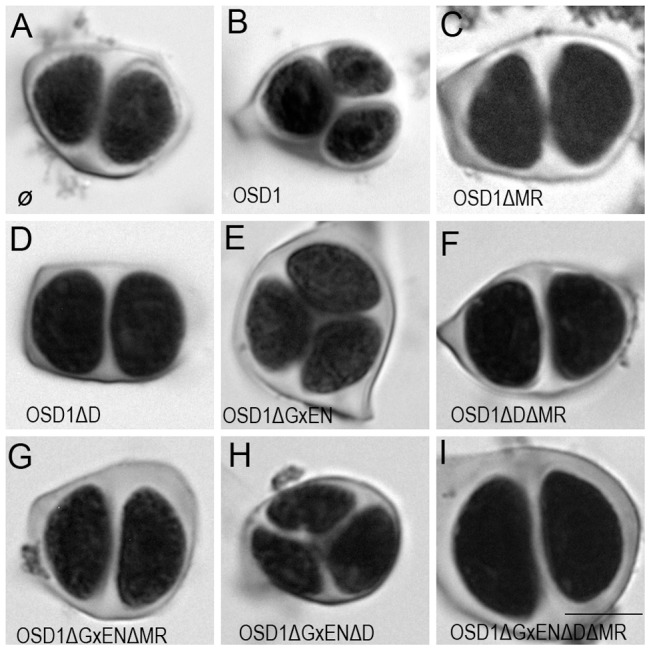
Complementation test of *osd1-3* by wild-type and mutated versions of OSD1. Male meiotic products stained by toluidine blue: (A) a dyad of spores from the *osd1-3* mutant. (B) A tetrad of spores from *osd1* complemented by the wild type *OSD1* gene. Note that one of the spores is out of focus because they are organized in a tetrahedron. (C to I) Male meiotic products from *osd1-3* transformed by versions of the *OSD1* gene with a GxEN-box mutation (OSD1ΔGxEN), a D-box mutation (OSD1ΔD), a MR-tail mutation (OSD1ΔMR) or combination of these mutations. Some versions induced complementation, with a majority of tetrads (E, H), while others did not restore tetrad formation (C, D, F, G, I). Scale bar = 10 µM.

**Figure 4:**
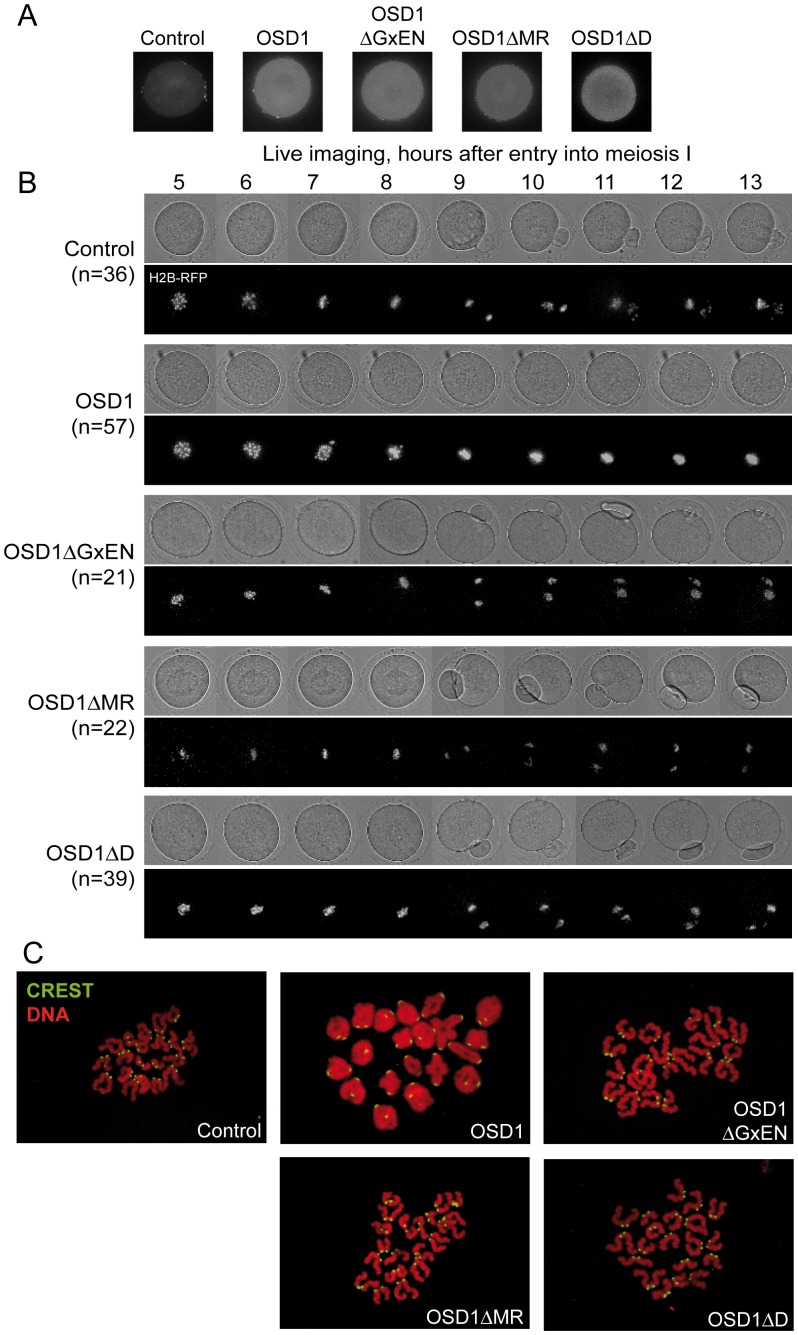
Expression of OSD1 in mouse oocytes provokes a metaphase I arrest. Germinal Vesicle (GV) stage mouse oocytes were injected with mRNA encoding the indicated OSD1 constructs. (A) Immunofluorescence on fixed oocytes in prometaphase I showing equal expression of the different *OSD1* constructs with anti-OSD1 antibody. (B) Histone H2B-RFP encoding mRNA was injected together with the indicated OSD1 mRNA to follow chromosome movements. Oocytes were induced to enter the first meiotic division in a synchronized manner, and followed by live imaging. Shown are images from the DIC channel and collapsed images of 8 z-sections of 2 µm to visualize H2B-RFP labelled chromosomes at selected time points. (C) Chromosome spreads at the end of the movie. Chromosomes were stained with propidium iodide (red), and kinetochores with CREST serum (green). Only in OSD1 injected oocytes chromosomes have not been separated in meiosis I.

To investigate the *in vivo* role of the APC/C interaction motifs revealed above, we created several versions of the genomic *OSD1* gene (including *OSD1* promoter and terminator) with a GxEN-box mutation (OSD1ΔGxEN, GxEN→AxAA), a D-box mutation (OSD1ΔD, RxxL→GxxV), a MR-tail mutation (OSD1ΔMR, MR→*) or combination of two or all of these mutations. None of these constructs modified the plant phenotype when introduced in wild type plants (data not shown). We then introduced them in the *osd1-3* mutant (Figure 3). The wild type genomic clone was able to restore normal meiosis (i.e formation of tetrads) of the *osd1-3* mutant (number of independent transformants n = 8, 8/8 100% tetrads). In contrast, OSD1ΔMR could not restore tetrad formation (n = 6, 0% tetrad) whereas OSD1ΔD barely complemented (n = 6, 0 to 15% tetrads). Albeit we cannot exclude that the introduced mutations destabilize the protein *in planta* (though the modified OSD1 proteins accumulated at equal level when expressed in yeast and mouse oocytes (Figure S3, Figure 4A)), these results indicate that the OSD1 D-box and MR-tail are important for OSD1 function. Correspondingly, the OSD1ΔDΔMR allele could not restore tetrad formation in *osd1-3* (n = 5, 0% tetrads). In contrast, OSD1ΔGxEN almost completely reverted the *osd1-3* mutant phenotype (n = 3, 82 to 93% tetrads), suggesting that the GxEN-box is not essential for the OSD1 function in *planta*. Strikingly, the OSD1ΔGxENΔD allele could complement *osd1* mutants (n = 4, 83 to 94% tetrads), showing that deleting the GxEN-box in OSD1ΔD restored OSD1 function. OSD1ΔGxENΔMR and OSD1ΔDΔGxENΔMR did not complement *osd1-3* (n = 2 and n = 4), showing that the MR tail is required in all situations (Figure 3).

### Expression of OSD1 in mouse oocytes provokes a metaphase I arrest

To further confirm that OSD1 is an APC/C inhibitor, we took advantage of the fact that - while OSD1 is not conserved in mammals - the APC/C and its activators are extremely well conserved. Thus, expression of OSD1 in a mammalian system - such as mouse oocytes- should equally interfere with APC/C activity and thereby disturb meiotic progression. OSD1 was stably expressed in mouse oocytes (Figure 4A). Oocytes injected with mRNAs encoding OSD1, but not control-injected oocytes, arrested at metaphase I with aligned chromosomes (visualized through simultaneous injection of H2B-RFP) (Figure 4B). Chromosome spreads reveal the presence of bivalents indicative of a metaphase I arrest (Figure 4C). This shows that OSD1 can inhibit the APC/C and prevent progression through meiosis I. Expression of OSD1ΔMR, OSD1ΔGxEN or OSD1ΔD (Figure 4A) did not provoke the metaphase arrest, showing that these three motifs are required for the APC/C inhibition by OSD1 in mouse oocytes (Figure 4B and 4C).

### Epistasis analysis between *OSD1*, CYCA1;2/*TAM* and *TDM*

**Figure 5:**
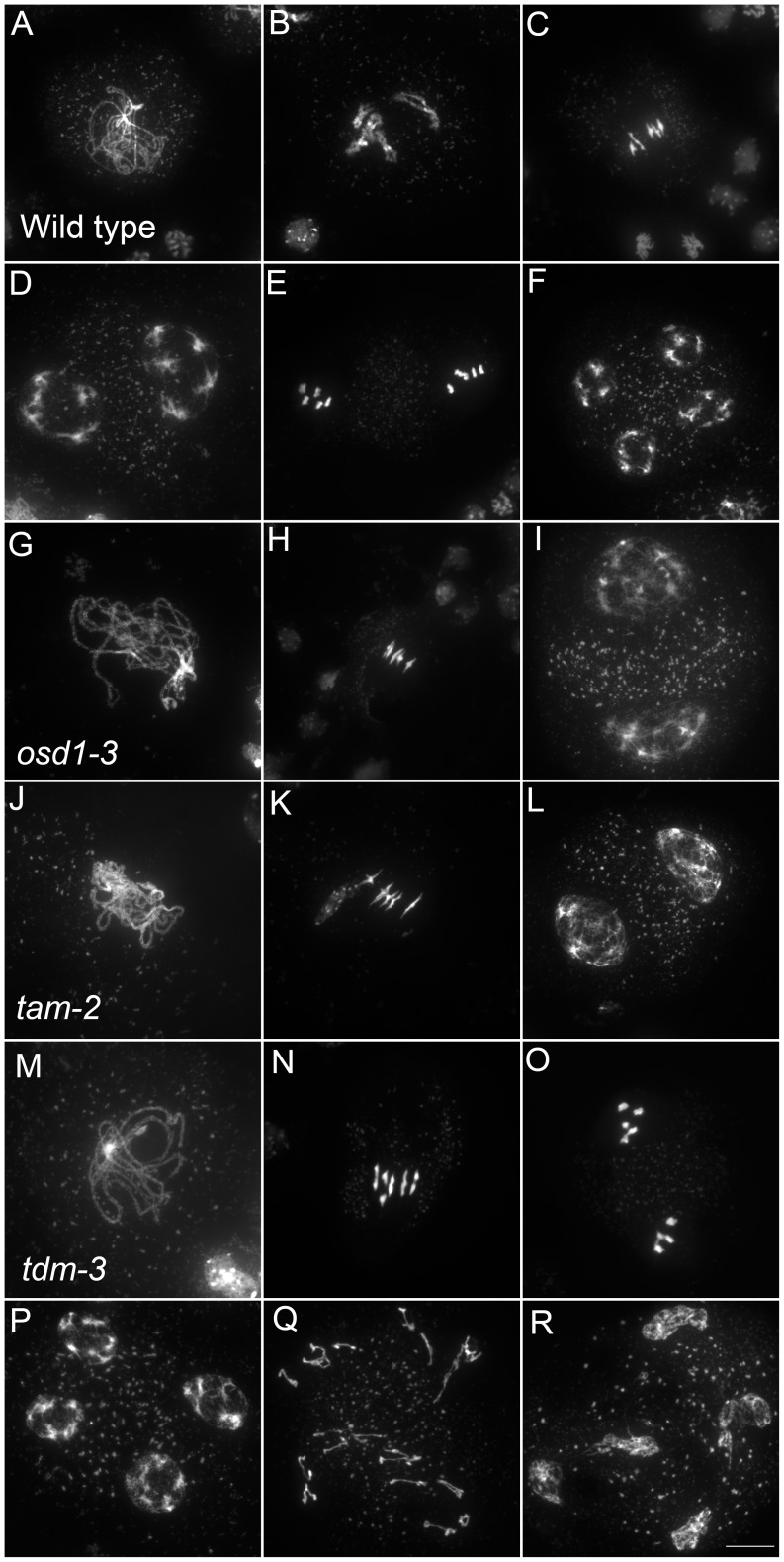
Meiotic chromosome spreads of wild type, *osd1-3*, *tam-2*, and *tdm-3* single mutants. (A to F) wild type. (A) pachytene, (B) diakinesis, (C) metaphase I, (D) telophase I, (E) metaphase II, (F) telophase II. (G to I) *osd1-3*. (G) Pachytene (H) metaphase I, (I) telophase I. (J to L) *tam-2*. (J) Pachytene, (K) Metaphase I, (L) telophase I. (M to R) *tdm-3*. (M) Pachytene (N) Metaphase I (O) metaphase II (P) telophase I (Q) aberrant third division (R) resulting telophase III. Scale bar = 10 µM.

**Figure 6:**
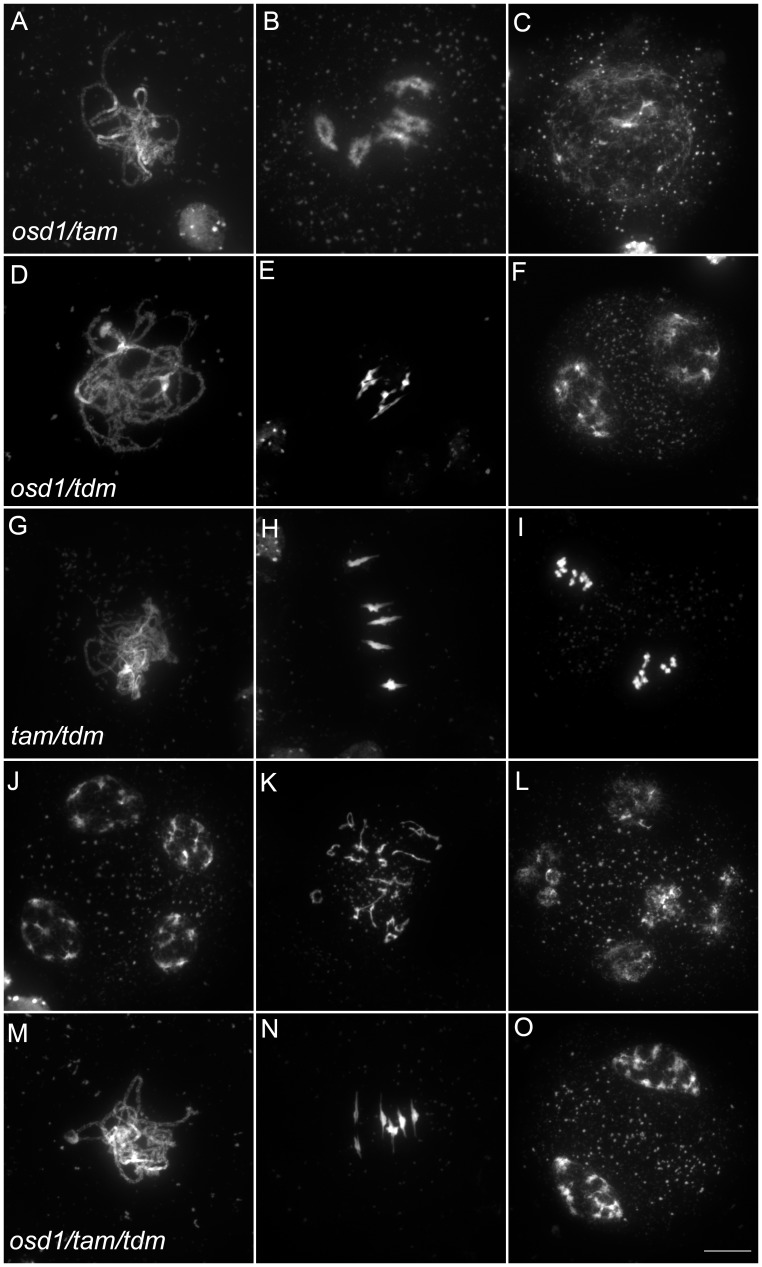
Epistasis analysis between OSD1, TAM, and TDM. Meiotic spreads of (A to C) *osd1-3/tam-2* double mutant, (D to F) *osd1-3/tdm-3* double mutant, (G to L) tam-2/tdm-3 double mutant and (M to O) *osd1-3/tam-2/tdm-3* triple mutant. Scale bar = 10 µM.

Only a few genes involved in control of the male meiotic cell cycle have been described in plants. Two mutants provoke premature exit before meiosis II – *osd1* and *cyca1;2/tam* [15]–[17]. In contrast, *tdm* mutation prevents exit from meiosis and provokes entry into a third round of division (meiosis III) after meiosis II [13], [20], [21] (Figure 5). The *tdm*-3 mutant is a newly described T-DNA allele which has the same phenotype as previously described *tdm* mutants [20], [21]. We studied the epistatic relationship between *osd1-3*, *tam-2* and *tdm-3* during male meiosis (Figure 6, Figures S4 and S5), completing prior work [13], [15]. As previously described with different alleles [13], the *tam-2/tdm-3* double mutant had the same phenotype as *tdm-3*, with a third division of meiosis (Figure 6 and Figure S4) and complete male sterility (Figure S4). In clear contrast, the double mutant *osd1-3/tdm-3* was male fertile (Figure S5) and meiocytes exited meiosis before meiosis II, like in the single *osd1-3* mutant (Figure 6 and Figure S4). Thus, depletion of TDM enables entry into meiosis II in *tam* but not *osd1* mutants. SMG7, which controls meiosis II exit through TDM [13], exhibits the same epistatic relationship with CYCA1;2/TAM and OSD1 as TDM (i.e *osd1-3* is epistatic to *smg7-1*; which is epistatic to *tam-2* [13], data not shown).

As we described previously with different alleles [15], meiocytes in the *osd1-3/tam-2* double mutant exit meiosis after a normal prophase I, without entering the first division (Figure 6 and Figure S4). In the triple mutant *osd1-3/tam-2/tdm-3* all male meiocytes progressed through meiosis I but arrested at telophase I, before cytokinesis (Figure 6 and Figure S4) leading to male sterility (Figure S5). Thus, mutating *TDM* allows *osd1-3/tam-2* to enter and progress into meiosis I. Notably, in contrast to the situation for the single *tam-2* mutant, mutating *TDM* in the *osd1-3/tam-2* double mutant does not completely suppress the *tam-2* defect, as the triple mutants are sterile and arrest at telophase I (no cytokinesis) whilst *osd1-3* plants are fertile and exit from meiosis after telophase I.

### CYCA1;2/TAM-CDKA;1 phosphorylates OSD1 *in vitro*

**Figure 7:**
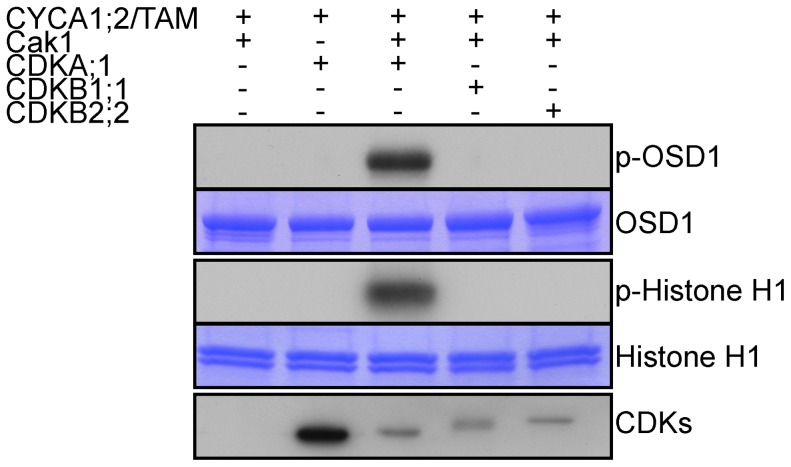
CDKA;1 is activated by CYCA1;2/TAM and phosphorylates OSD1 *in vitro*. HisMBP-CYCA1;2/TAM was co-expressed with StrepIII-CDKA;1 and GST-Cak1 in *E. coli*. HisMBP-CYCA1;2/TAM proteins were purified by means of a Ni-NTA column. HisMBP-OSD1 (Top) or Histone H1 (middle) were used as substrates in the kinase reaction. Coomassie blue staining of the gel shows equal loading of the respective substrate. StrepIII-CDKs co-purified with HisMBP-CYCA1;2/TAM were detected with strep-tactin HRP, showing identical amount of the kinases in each reaction (bottom).

OSD1 contains 7 predicted CDK phosphorylation sites (4 [S/T]P and 3 [S/T]Px[R/K]), five of them being well conserved (Figure S1), suggesting that it could be the target of a CDK. Co-precipitation assays from *E. coli* expressed proteins showed that CYCA1;2/TAM binds to all three kinases: CDKA;1, CDKB1;1 and CDKB2;2, although CDKA;1 appears to have a higher affinity to CYCA1;2/TAM than the others (Figure 7). However, a subsequently performed kinase assay revealed that only CYCA1;2/TAM-CDKA;1 but not CYCA1;2/TAM-CDKB1;1 or CYCA1;2/TAM-CDKB2;2 is active against both OSD1 and the generic substrate histone H1. These results suggest a regulatory interaction between CYCA1;2/TAM-CDKA;1 and OSD1 in meiosis.

### CYCA1;1 has no apparent meiotic function

CYCA1;2/TAM is likely not the sole cyclin promoting meiosis progression because male meiosis continues until the end of the first division in *tam-2* mutants. Female meiosis is less affected than male meiosis, as 60% of the female gametes are haploid, produced by a complete meiosis [15]. Among the 10 *Arabidopsis* A-type cyclins, CYCA1;1 is the most similar to CYCA1;2/TAM [26] and therefore a good candidate to have similar, possibly redundant, functions to those of CYCA1;2/TAM. We thus characterized an *Arabidopsis* line carrying a T-DNA insertion in *CYCA1;1* (*cyca1;1-1*, see M and M) which displayed no defects during meiosis and produced normal diploid progeny. Further, the double mutant *cyca1;1-1/tam-2* exhibited the same meiotic phenotype and produced similar frequencies of haploid/diploid gametes as *tam-2* (70% triploids and 30% tetraploids among the progeny of selfed double mutant). Hence, CYCA1;1 does not appear to have a meiotic function.

### Expression of a non-destructible CYCA1;2/TAM provokes the entry into an aberrant third meiotic division, mimicking the *tdm* phenotype

**Figure 8:**
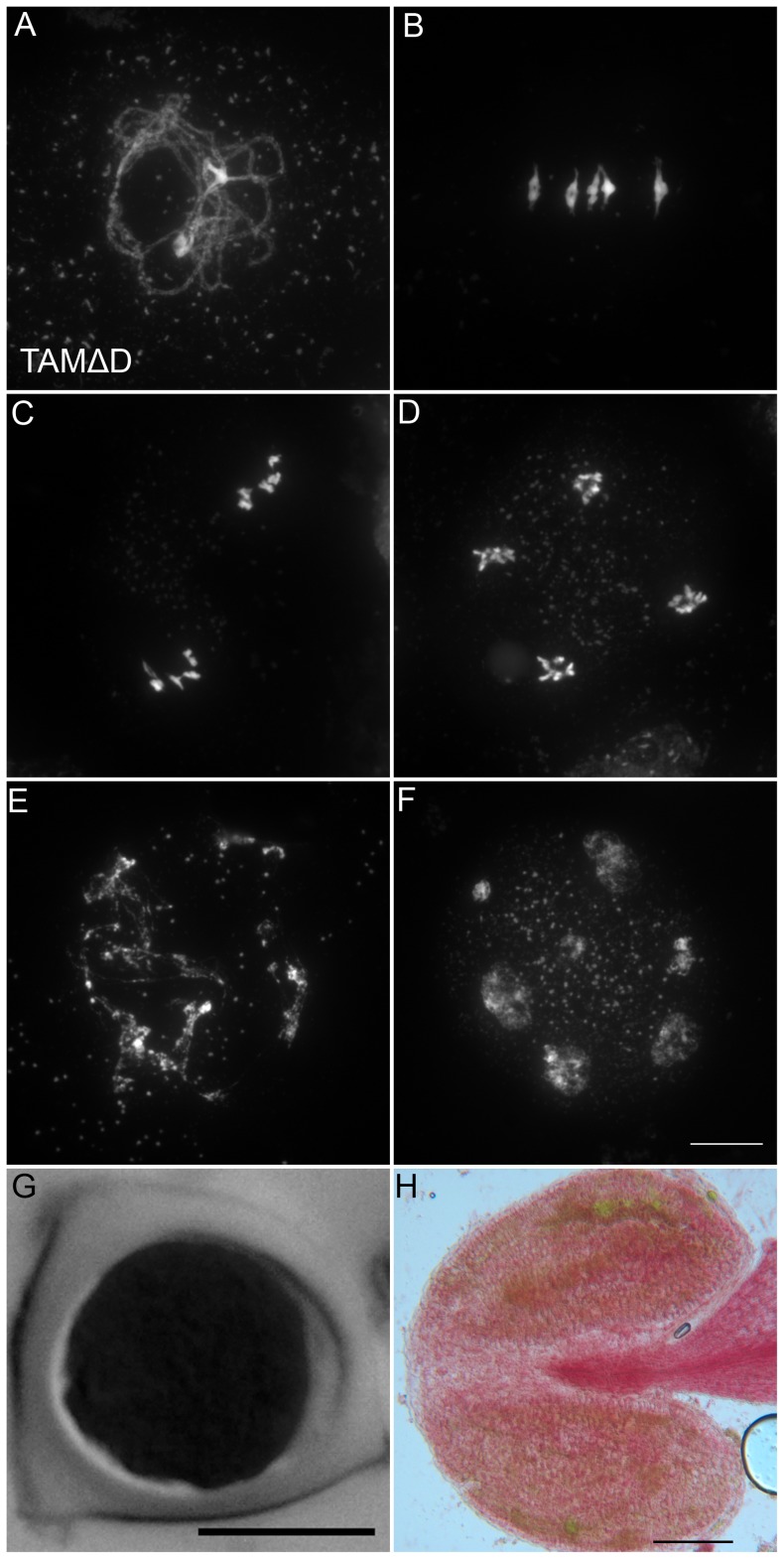
TAMΔD provokes the entry into a third meiotic division. (A to F) Meiotic spreads of wild type plants transformed by TAMΔD. (A) Pachytene. (B) Metaphase I. (C) Metaphase II. (D) late anaphase II (E) Aberrant third division (F) Resulting telophase III with seven nuclei. (G) Meiotic product stained by toluidine blue. Scale bar = 10 µM. (H) Alexander staining of an anther, showing the complete absence of pollen grains (Compare to Figure S5A). Scale bar = 100 µM.

**Figure 9:**
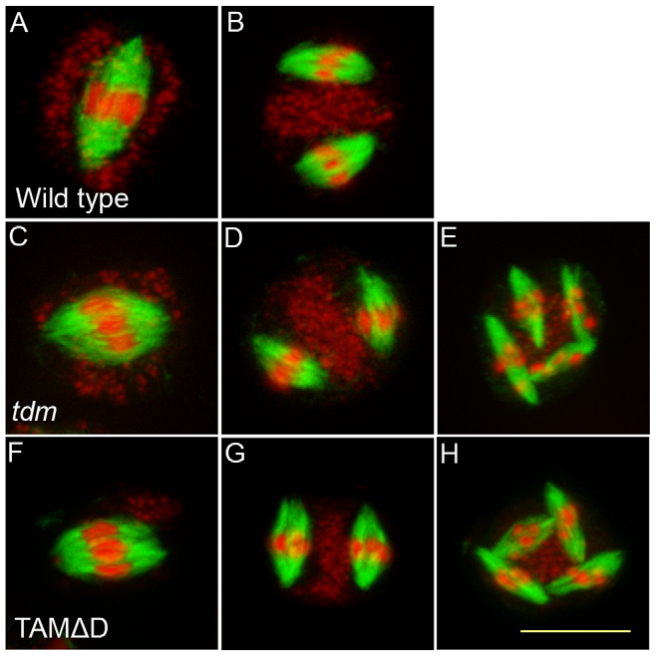
Four spindles form at meiosis III in TAMΔD plants. Immunolocalization of tubulin during meiosis. DNA appears in red and tubulin in green. (A and B) Wild type. One spindle is visible at metaphase I (C) and two at metaphase II (B). (A to C) *tdm*. After regular meiosis I (C) and meiosis II (D), *tdm* meiocytes enter a third division of meiosis with the formation of four spindles (E) [13]. (F to H) Wild type transformed by TAMΔD. Like in *tdm*, TAMΔD meiocytes perform a third meiotic division with the formation of four spindles (H).

**Figure 10:**
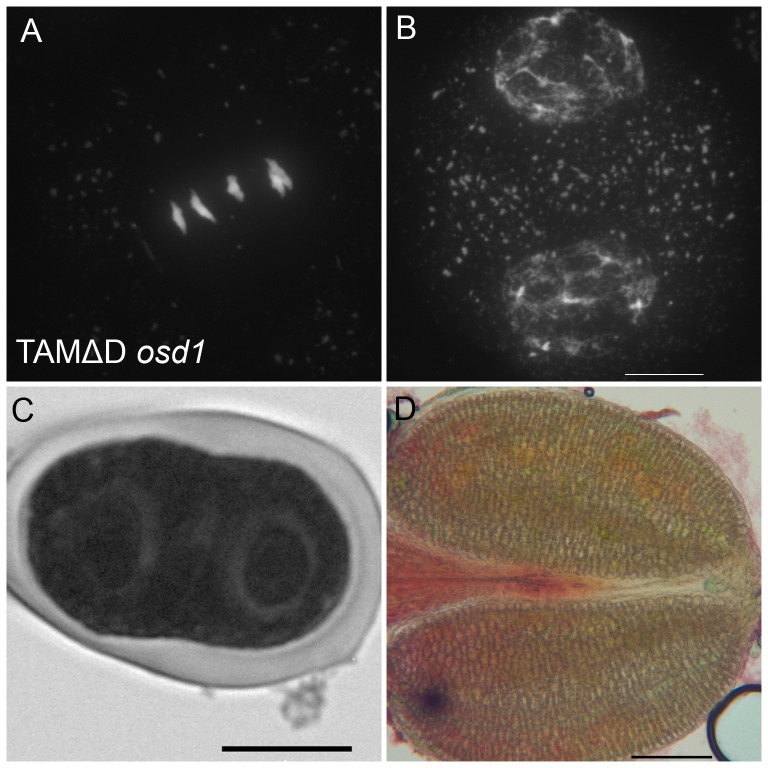
TAMΔD in *osd1*. (A and B) Meiotic spreads of *osd1-3* transformed by TAMΔD. (A) Metaphase I. (B) Telophase I. (C) Meiotic product stained by toluidine blue. Scale bar = 10 µM. (D) Alexander staining of an anther, showing the complete absence of pollen grains. Scale bar = 100 µM.

Like many cyclins, CYCA1;2/TAM possesses a D-box [18], a domain essential for cyclin destruction by the APC/C. We thus created a genomic version of the *CYCA1;2/TAM* gene, including endogenous promoter and terminator, with a D-box mutation (TAMΔD, RxxL→GxxV). The corresponding wild type construct rescued the *tam-2* meiotic defect (n = 5, 100% tetrads). In contrast, the introduction of TAMΔD in either wild type or *tam-2* mutant (n = 8), generated a dominant effect on male and female meiosis. Plants containing the TAMΔD transgene produced only monads and were completely male and female sterile (Figure 8). No somatic phenotype was observed, strongly suggesting that CYCA1;2/TAM functions specifically at meiosis. Meiotic chromosome spreads showed that meiosis in TAMΔD plants progressed through meiosis I and meiosis II, up to telophase II ((Figure 8A–8D). But then, meiosis entered into an aberrant third division of meiosis, with stretched chromosomes dispersed throughout the cell, and no cytokinesis (Figure 8E–8F). Immunolocalization of tubulin, confirmed that meiocytes expressing TAMΔD entered a third meiotic division, with the formation of four spindles (Figure 9), like previously shown for the *tdm* mutant [13]. When TAMΔD was introduced in *tdm-3* (n = 5), meiosis progressed through meiosis I and meiosis II, and entered into the third division of meiosis typical of *tdm* or TAMΔD (not shown). In contrast, when TAMΔD was introduced into *osd1-3* (n = 5), meiosis progressed through meiosis I and arrested at telophase I, without entering meiosis II (Figure 10). Unlike single *osd1-3*, cytokinesis did not occur (Figure 10C), the meiotic product did not develop into pollen grains, and the plants were sterile (Figure 10D).

### OSD1 and UVI4 are synthetically essential for female gametogenesis and somatic growth

When *UVI4*, *the OSD1* paralogue, is mutated, an increase of somatic endoreduplication and no meiotic phenotype is observed [27]. It has also been recently shown that mutation of *OSD1*, in addition to its meiotic consequences, triggers ectopic endomitosis [19]. To determine the interaction between OSD1 and UVI4, we aimed to produce a double *osd1/uvi4* mutant. However no double mutant was recovered from self-pollinating populations of *osd1-1*+/− *uvi4*+/−, *osd1-2+/− uvi4+/−*, or *osd1-2+/− uvi4−/−* (92 double mutants expected in total, Table S1). This distortion indicates that the mutation of both *OSD1* and *UVI4* leads to gametophyte and/or embryo lethality. Reciprocal crosses between *osd1-2+/− uvi4+/−* or *osd1-2+/− uvi4−/−* plants and wild-type plants showed that transmission of *osd1* and *uvi4* through male gametophyte is regular but that the transmission of the double *osd1/uvi4* mutant allele through female gametophyte is reduced by 80% (Table S1).

**Figure 11:**
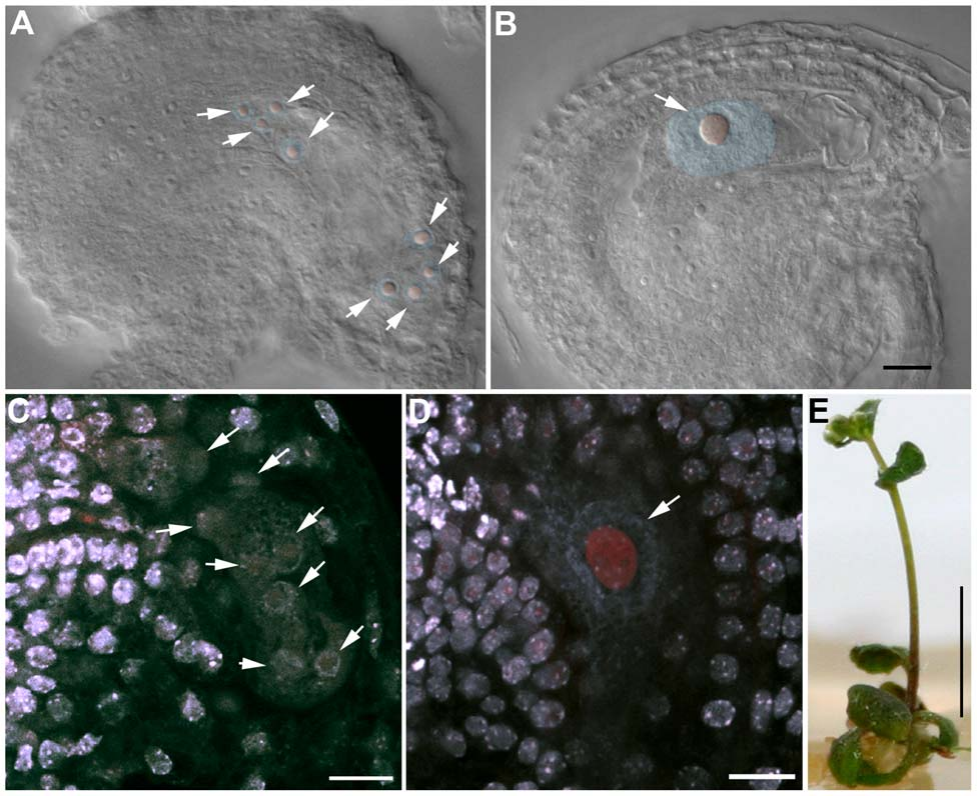
OSD1 and UVI4 are synthetically essential for female gametogenesis and somatic growth. (A and B) Cleared female gametophyte. Nuclei and nucleoli have been artificially highlighted in blue and red, respectively. (A) Wild type at the 8 nuclei stage. A nucleolus is visible in the center of each nucleus (arrows) (B) A female gametophyte in the *uvi4+/− osd1-2*+/− plant at a comparable stage, showing a single giant nucleus with a massive nucleolus. (C to D) Double staining of female gametophytes with DAPI and propidium iodide. The DNA is stained in blue and the nucleoli appear in red. (C) Wild type at the 8 nuclei stage (arrows) (D) One female gametophyte in *uvi4+/− osd1-2*+/− containing a single large nucleus with a great amount of DNA, and a large nucleolus. Scale bar = 10 µM. (E) A 5 weeks old *osd1/uvi4* double mutant. Scale bar = 5 mm.

We observed female gametophyte development in *osd1-2+/− uvi4−/−* plants, in which 50% of the gametophytes are expected to inherit the double *osd1/uvi4* mutation. Wild type female gametophyte development includes three haploid mitotic events, leading to the formation of eight nuclei (Figure 11A and 11C). In *osd1-2+/− uvi4−/−*, 58% (n = 371) of the gametophytes showed wild type-like development, the others being blocked at a 1 cell (37%) or 2 cell stage (5%) (Figure 11B). These arrested cells had a very large nucleus, with an increased DNA content (compare Figure 11C and 11D), suggesting a defect in mitotic cell cycle. Both genetic and cytological data suggested that some *osd1/uvi4* female gametophytes may be viable, prompting us to look further for double mutant plants. Among approximately 13,000 seeds of an *osd1-2+/− uvi4−/−* plant sown *in vitro*, 25 very abnormal plants were identified and confirmed by genotyping to be *osd1-2−/− uvi4−/−*. These plants, due to strongly affected growth, measured at most 2 cm after 5 weeks (Figure 11E), while wild type plants of the same age were fully developed and about 30 cm high. Altogether, these results show that OSD1, beyond its meiotic function has an essential function, redundantly with UVI4, in gametophyte and somatic growth.

## Discussion

### OSD1, CYCA1;2/TAM, and TDM form a regulatory network that controls the key transitions of meiosis

Our and previously published data suggest that a functional network between OSD1, CYCA1;2/TAM and TDM controls three key transitions of meiosis (prophase-meiosis I, meiosis I-meiosis II and meiosis II-exit) : (i) OSD1 and CYCA1;2/TAM act in a synergetic manner to promote the transition from prophase to meiosis I, as the double mutant fails to enter meiosis I. TDM, appears to repress this transition as its mutation restores the entry into meiosis I of the *osd1-3/tam-2* double mutant. (ii) OSD1 and CYCA1;2/TAM are crucial for the meiosis I-meiosis II transition as both single mutant exit meiosis before meiosis II. TDM also appears to repress this transition as its mutation allows the *tam-2* mutant to enter meiosis II. Interestingly, OSD1 appears to be absolutely essential for the entry into meiosis II as *osd1* mutants never entered this phase in all the backgrounds we tested. (iii) TDM and CYCA1;2/TAM are also involved in the exit from meiosis II, to prevent entry into a third meiotic division. Indeed, the mutation of TDM or the expression of a non-destructible version of CYCA1;2/TAM provokes the entry into a third division of meiosis. It is unclear if OSD1 could also be involved in this transition, as *osd1* mutants never reach this stage. Further investigation is required to understand how this network is fine tuned to allow entry into a division after prophase and after meiosis I but to allow exit after meiosis II.

### OSD1 is a meiotic APC/C inhibitor

Control of APC/C activity is fundamental for regulation of cell cycle progression. The APC/C is an E3 ubiquitin ligase that triggers the degradation of multiple proteins, including cyclins, at meiosis and mitosis [3]. Several APC/C inhibitors with crucial functions at mitosis or meiosis have been identified in various eukaryotes (e.g. EMI1 and EMI2/ERP in vertebrates, Mes1 in *S. pombe*, Mnd2 and Acm1 in *S cerevisiae*), but these proteins are not conserved between kingdoms. An independent study recently showed that OSD1 negatively regulates the APC/C to prevent endo-mitosis during somatic development [19]. Here we propose that OSD1, similar to Mes1 in *S. pombe* and Emi2 in vertebrates, promotes meiotic progression through APC/C inhibition. Indeed, OSD1 interacts directly with activator subunits of the APC/C, as shown by both TAP and Y2H experiments (ours and Iwata et al results [19]). In addition, expression of OSD1 in mouse oocytes provokes meiotic arrest at metaphase I, consistent with APC/C inhibition.

Similar to Mes1, OSD1 contains three APC/C interaction domains, a D-box, a GxEN/KEN-box and a MR-tail. Both the interaction with the APC/C and the *in planta* meiotic function of OSD1 are dependent on its D-Box domain and its MR tail, suggesting that OSD1 inhibits the APC/C through direct binding with its active site or by sequestering its activators. Remarkably, the OSD1 GxEN-box is not required for OSD1 function, but its mutation allows the OSD1 protein mutated in its D-Box to fulfill its function. However, the three domains (D-box, GxEN/KEN-box and a MR-tail) are required to provoke meiotic arrest when OSD1 is overexpressed in mouse oocytes.

The modulation of the cell cycle machinery that permits the entry into a second division without an intervening replication at meiosis seems to be fulfilled in various eukaryotes thanks to apparently evolutionary unrelated APC/C inhibitors, Mes1 in *S. pombe*, Emi2 in vertebrates and OSD1 in plants. Interestingly, OSD1 and Emi2 both have a paralogue in their respective genomes, UVI4 and Emi1 respectively, that play roles in the mitotic cell cycle through APC/C regulation [3], [28]. However, contrary to Mes1, OSD1 has also a somatic function as revealed by the mitotic phenotype of the single *osd1* mutants [19] and the strong gametophytic and somatic defects of the *osd1/uvi4* double mutant.

### What could be the function of TDM?

The molecular function of TDM is unknown. However, four TPR domains (AA 61–191) were predicted with high probability in TDM (P = 8.0E-12) [29]. Further, using remote similarity searches via HHpred [30], we found that CUT9 (a TPR-containing APC/C component, appeared as the first hit (Protein Data Bank entry 2xpi_A, E = 0.00095). APC16, another TPR-containing APC/C component also appeared among the first hits (3hym_B, E = 0,0022). This raised the possibility that TDM may interact with or may be a component of the APC/C, and thus promotes meiotic exit via APC/C-mediated cyclin destruction.

### What could be the function of CYCA1;2/TAM?

CDKA;1 appears to be a major cyclin-dependent kinase that drives meiotic progression in plants [12], [13]. However, the cyclin(s) forming (with CDKA;1) the predicted core cyclin/CDK meiotic oscillator has/have not been identified yet. Two cyclins have been shown to have an essential role at meiosis, CYCA1;2/TAM and SDS. However, SDS has been shown not to affect meiotic progression, but to regulate the choice of the partner of homologous recombination [31], [32]. CYCA1;2/TAM being the sole known cyclin whose mutation affects meiotic progression, appeared to be a good candidate to fulfill part of this function. Indeed the fact that *tam* null mutants exit prematurely from meiosis supports this hypothesis. We also showed here that TAM is an active cyclin as it can form an active complex with CDKA;1. However, prior evidence suggests that CYCA1;2/TAM may not be the core CDK oscillator that drives meiotic divisions [13]. Correspondingly, we showed here that the expression of a non-destructible CYCA1;2/TAM does not provoke a meiotic arrest at metaphase/anaphase I as may be expected for the core CDK oscillator, but induces entry into a third division. Strikingly, the phenotypes induced by the *tdm* mutation or the expression of non-destructible CYCA1;2/TAM appear identical, suggesting that TDM and TAM act in an antagonist manner to promote and prevent exit from meiosis, respectively. In addition, *tdm* is epistatic to *tam-2*. Two hypotheses may account for these results (Figure S6). (i) CYCA1;2/TAM could be a negative regulator of TDM, which itself promotes meiotic exit, maybe through APC/C activation. (ii) Alternatively, another cyclin(s) (distinct from CYCA1;1 as shown here) might, together with CYCA1;2, promote directly meiosis progression in a dose-dependent manner. The function of TDM could be to negatively regulate these cyclins, possibly through activation of the APC/C that would clear the cell from the remaining cyclins at the end of the meiotic program. Further work is required to discriminate between these hypotheses (Figure S6).

We also showed that the CYCA1;2/TAM-CDKA;1 complex phosphorylates OSD1, at least in *vitro*. This suggests that TAM could regulate OSD1 to prevent precocious meiotic exit. Alternatively, phosphorylation by TAM could inactivate OSD1 and thus allow exit from meiosis I. Interestingly, the activity and stability of Emi2/Erp1 - the vertebrate meiotic APC/C inhibitor - is regulated by phosphorylation [33], [34]. Further functional analysis of the OSD1 putative phosphorylation sites is required to establish the role of this CYCA1;2/TAM-CDKA;1-mediated phosphorylation in meiotic cell cycle progression.

These are the early days of meiotic cell cycle studies in plants, and already a complex regulatory network has emerged. Further studies are required to understand the control of meiotic progression in plants, and notably one of the next important goals is to establish which cyclin(s) constitutes the core meiotic progression oscillator and which activators of the APC/C (among the five putative CDC20 and three CDH1) are involved in this complex variation of the cell cycle.

## Materials and Methods

### Growth conditions and genotyping

*Arabidopsis* plants were cultivated in greenhouse as previously described [35] or *in vitro* on Arabidopsis medium [36] at 21°C, under a 16-h to 18-h photoperiod and 70% relative humidity.

For epistasis studies, we used *osd1-3*, *tam-2* and *tdm-3*, alleles that are all in the same genetic background (Col-0), to prevent any genetic complication. The T-DNA of the *tdm-3* mutant (SALK_034202) is inserted in the second exon (ATG+609 pb). In the *cyca1;1-1* (pst18025), the T-DNA insertion is in the fifth exon (ATG+1415 pb). The *tdm-3* and *cyca1;1-1* mutants were genotyped by PCR by two primer pairs. The first pair is specific to the wild type allele and the second to the left border of the inserted sequence. *tdm-3*: N534202U (5′- GGAGATCGAGTTGATAGTGC-3′) & N534202L (5′-ATACTAGGGAACTTGGGCT-3′); N534202U & LbB1 (5′-GCGTGGACCGCTTGCTGCAACT-3′); *cyca1;1-1* : pst18025U (5′-TTGATTTGCTTGGTATTGCAG-3′) & pst18025L (5′–TGGTCGTCTTGTTGGGTCTAG-3′); pst18025L & Ds5-2a (5′-TCCGTTCCGTTTTCGTTTTTTAC-3′). The primers used to genotype *tam-2*, *osd1-1*, *osd1-2 and osd1-3* were previously described [15], [16], [28]. The *uvi4* mutant (*pym*) [27] was genotyped by CAPS using the primers: pymU (5′-GGAGTGCTCTTCATTTTCTG-3′), pymL (5′-TCTCATTTTGGATTTGTCTG-3′) and the restriction enzyme BsuRI (419 pb+158 pb for the mutant versus 286 pb+133 pb+158 pb for the wild type allele).

### Sequence analysis

HHpred searches were performed on user defined query alignment, without automatic PSI-BLAST enrichment of the query set and by using otherwise default settings [29], [30]. The alignment of OSD1 proteins was performed with T-Coffee using default settings [29], [37].

### Tap-Tag/Y2H

Tandem affinity purification constructs were generated and purified as described previously [38]. UVI4, OSD1, CDC20.1, CDC20.3, CCS52A1, CCS52A2 and CCS52B tandem purified baits were separated by SDS-PAGE and probed with an anti-OSD1 antibody after western blotting. Yeast 2-hybrid interaction testing using OSD1 as bait (pDEST32) with different APC/C subunits as prey (pDEST22) was performed by mating, as described previously [28]. For mutant allele interaction screening, *OSD1* mutant alleles were tested as bait (pDEST32) against CCS52A1 as prey (pDEST22) and introduced in the yeast PJ69-4a strain by cotransformation.

### Mouse oocytes

GV stage oocytes were harvested from 9–16 weeks old CD-1 (Swiss) mice (Janvier), injected and analyzed by live imaging essentially as described in [39]. H2B-RFP (gift from Z. Polanski, Cracow, Poland) mRNA was synthesized with the T3 mMessage Machine kit (Ambion) according to the manufacturer's instructions. For live imaging, a motorized inverted Nikon TE2000E microscope (Plan APO 20x/0,75NA objective) with PrecisExite High Power LED Fluorescence (LAM 1: 400/465, LAM2: 585), equipped with a temperature chamber (Life Imaging Services), Märzhäuser Scanning Stage, CoolSNAP HQ2 camera, and controlled by Metamorph software was used. Timepoints were taken every 20 minutes. Images were treated with ImageJ software. Immunofluorescence studies on formaldehyde fixed prometaphase I oocytes with anti-Osd1 antibody (1∶150), and chromosome spreads of metaphase II oocytes were performed as described in [39].

### Phosphorylation

OSD1 cDNA was amplified by sequential PCR first using attB1Ad-OSD1_s (5′-AAAAAGCAGGCTTCATGCCAGAAGCAAGAGATCG-3′), attB2Ad-OSD1_as (5′-AGAAAGCTGGGTCTCATCGCATAGTCATTAAAGTCCG-3′) followed by attB1 adapter primer (5′-GGGGACAAGTTTGTACAAAAAAGCAGGCT-3′), attB2 adapter primer (5′-GGGGACCACTTTGTACAAGAAAGCTGGGT-3′). The PCR product was cloned, by Gateway (Invitrogen), into the pDONR223 vector (Invitrogen). A recombination reaction was performed between the resulting entry clone and a destination vector pHMGWA [40]. *E. coli* SoluBL21 cells (AMS Biotechnology) were transformed with the resulting destination clone, and grown in LB medium containing 100 µg/ml ampicillin until OD_600_ = 0.6 at 37°C. The culture was transferred to 18°C and grown for 30 min. The production of the fusion protein was induced by adding 0.3 mM IPTG (isopropyl-β-d-thiogalactopyranoside,Thermo scientific) overnight at 18°C. Cells were harvested by centrifugation and re-suspended in Ni-NTA binding buffer (50 mM NaH_2_PO_4_, 100 mM NaCl, 10%(v/v) glycerol, 25 mM imidazole, pH 8.0), and lysed by sonication. After addition of Triton X-100 to 0.2%(w/v), the cell slurry was incubated at 4°C then clarified by centrifugation. The supernatant was passed through a column packed with Ni-NTA resin (Qiagen), which was washed sequentially with Ni-NTA binding buffer followed by kinase buffer (50 mM Tris-HCl, pH 7.5, 10 mM MgCl_2_, 1 mM EGTA) containing 150 mM NaCl, and eluted with kinase buffer containing 150 mM NaCl and 200 mM imidazole. CYCA1;2/TAM cDNA was cloned into pHMGWA as described above by using primers attB1Ad-TAM_s (5′-AAAAAGCAGGCTTCATGTCTTCTTCGTCGAGAAATCTATC-3′) and attB2Ad-TAM_as (5′-AGAAAGCTGGGTCTCAGAGGAAAAGCTCTTGCG-3′) followed by attB1 adapter primer and attB2 adapter primer. CYCA1;2/TAM-CDK complexes were prepared from *E. coli* as described [14]. Kinase reactions were performed as described in [14] using kinase buffer. In the case of OSD1, kinase buffer containing 150 mM NaCl was used.

### Cytology

Observation of final male meiotic products and chromosomes spreads were carried out as previously described [31], [41] and observed with a ZEISS AxioObserver microscope. Observation of developing ovule by DIC and confocal microscopy was performed as described by Motamayor et al [42]. Alexander staining was performed according to [43].

### Immulocalization of tubulin

Inflorescence were fixed in ethanol∶acetic acid (3∶1) and digested for 1 h as described in [41]. Meiocytes were squashed and immobilized on polysin slides as described in [44], digested again for 30 min at 37°C in the digestion medium described in [41] and subsequently incubated one hour in PBS 1% Triton at room temperature. After 2 rinses with PBS 0.1% Triton, slides were incubated overnight at 4°C in primary antibodies (mouse anti-tubulin (Sigma T5168) diluted at 1/300 in PBS, 1% BSA, then washed in PBS, 0.1% Triton 5 times for 10 min. After 2 h of incubation at 37°C with the secondary antibodies in PBS 1% BSA, slides were washed in PBS 0.1% Triton 5 times for 10 min and mounted in Vectashield antifade medium (Vector Laboratories) with 80 µg/ml propidium iodide. Images were acquired with Zeiss Apotome.

### OSD1 antibody

An anti-OSD1 antibody was raised against a full-length recombinant protein as described in [35].

### Directed mutagenesis constructs and plant transformation

OSD1 and CYCA1;2/TAM genomic fragment were amplified by PCR using OSD1 U (5′-CATATAAGCCTTGACCCTCTTTC-3′), OSD1 L (5′-AGAAACCACCGAACTTGTGAAGA-3′) and TAM U (5′-CCAGTCACCACAATACACAC-3′), TAM L (5′-GCGGTTTGGGTTGGTTTTTGTTT-3′). The amplification for OSD1 covered 1603 nucleotides before the ATG and 170 nucleotides after the stop codon. The amplification for CYCA1;2/TAM covered 1495 nucleotides before the ATG and 493 nucleotides after stop codon. The PCR product was cloned, by Gateway (Invitrogen), into the pENTR vector (Invitrogen), to create pENTR-OSD1 and pENTR-TAM, respectively, on which directed mutagenesis was performed using the Stratagene Quickchange Site-Directed Mutagenesis Kit. The mutagenic primers used to generate the OSD1ΔD, OSD1ΔGXEN and OSD1ΔMR mutations were (5′-GCCTTCTTGGTATCCA**G**GAACACCT**G**TACGCGACATAAC-3′), (5′- GATTGCCACAGG**C**AAGAG**C**G**GC**TATGCCCATAG-3′) and (5′- GGTGCGGACTTTAATGACT**TA**GCGATGATCTTTACTTAGG-3′) respectively. The mutagenic primers used to generate the TAMΔD were (5′- GTTGGAAACCGT**GGT**GCTCCC**GTC**GGCGACATCACAAATC-3′). To generate binary vectors for plant transformation, an LR reaction was performed with the binary vector for the Gateway system, pGWB1 [45]. The resulting binary vectors, pOSD1, pOSD1ΔD, pOSD1ΔGXEN, pOSD1ΔMR, pOSD1ΔDΔGXEN, pOSD1ΔDΔMR, pOSD1ΔDΔGXENΔMR and pTAM, pTAMΔD, were transformed using the Agrobacterium-mediated floral dip method [46], on plant populations segregating for the *osd1-3*, *tam-2* or *tdm-3* mutation. Transformed plants were selected on agar plates containing 50 mg/L kanamycin for OSD1 constructs and 20 mg/L hygromycin for CYCA1;2/TAM constructs, respectively.

## Supporting Information

Figure S1 T-Cofee alignment of OSD1 and UVI4 plant proteins. Identical or similar residues conserved in more than 50% of the proteins are shaded in black and grey, respectively. Red rectangles indicate the GxEN/KEN-box, D-box and MR-tail. Stars point to putative phosphorylation sites.
(RTF)

Figure S2 The anti-OSD1 antibody is specific. Probing of UVI4 and OSD1 TAP elutions with anti-OSD1 antibody shows it recognizes specifically OSD1.
(TIF)

Figure S3 Mutated version of OSD1 are stably expressed in yeast. Protein extracts [47] of yeasts transformed with wild type or mutated version of *OSD1* were probed with anti-OSD1 antibody. Wild type or mutated version of OSD1 showed similar expression levels.
(TIF)

Figure S4 Meiotic product stained by toluidine blue. (A) *tam-2*. (B) *tdm-3*. (C) *osd1-3/tdm-3*. (D) *osd1-3/tam-2*. (E) *tam-2/tdm-3*. (F) *osd1-3/tam-2/tdm-3*. Scale bar = 10 µM.
(TIF)

Figure S5 Alexander staining of anthers. Viable pollen grains are stained in red. (A) Wild type. (B) *osd1-3* (C) *tam-2*. (D) *tdm-3*. (E) *osd1-3/tam-2*. (F) *osd1-3/tdm-3*. (G) *tam-2/tdm-3*. (H) *osd1-3/tam-2/tdm-3*. Scale bar = 100 µM.
(TIF)

Figure S6 Two alternative models for the OSD1, CYCA1;2/TAM and TDM functional network.
(TIF)

Table S1 Genetic analysis of *osd1/uvi4* transmission.
(PDF)
