# Supplementary material for: OSD1 Promotes Meiotic Progression via APC/C Inhibition and Forms a Regulatory Network with TDM and CYCA1;2/TAM
Source: PLoS Genet. 2012 Jul 26;8(7):e1002865. doi: 10.1371/journal.pgen.1002865 (PMC3406007; doi:10.1371/journal.pgen.1002865)
Supplement: Figure S1 — T-Cofee alignment of OSD1 and UVI4 plant proteins. Identical or similar residues conserved in more than 50% of the proteins are shaded in black and grey, respectively. Red rectangles indicate the GxEN/KEN-box, D-box and MR-tail. Stars point to putative phosphorylation sites. (RTF) [file pgen.1002865.s001.rtf]

                                         10        20        30        40        50        60        70        80        90       100                  
                                ....|....|....|....|....|....|....|....|....|....|....|....|....|....|....|....|....|....|....|....|
Arabidopsis_thaliana_OSD1       MPEARDRTERPVDYSTIFANRRRH---GILLDEP-DSRLSLIESPVNPD------------------I--------GSIG---------GTG-GLVRGNF 60  
Arabidopsis_thaliana_UVI4       MPEARDRIERQVDYPAAFLNRRSH---GILLDEP-ATQHNLFGSPVQRV------------------PS-EATGGLGSIGQ----GSMTGRG-GLVRGNF 72  
Arabidopsis_lyrata_XP_00287644  MPEARDRIERPVDYPAIFVNRRSN---GVLLDEP-DSRLSLIESPVNPE------------------T--------GSMGR----GSLVGTG-GLVRGNF 65  
Arabidopsis_lyrata_JGI903574    MPEARDRIERPVDYPAAFLNRRSH---GILLDEP-ATHHNLFGSPVQRV------------------PS-EAT-GLGSVGQ----GSMMGRG-GLVRGNF 71  
Brassica_rapa_EX107108          MPEARDRRERSVDYPAAFLNRRSH---GILLDES-PLR-----SPVQRL------------------PS----------SE----SLVFGRG-GFARGNL 58  
Brassica_rapa                   MAEARDRLEKPVDYAAIFANRRSH---GVLLDEP-EAGLGVLEHPVRRL------------------PS-------GS--R------VYPQP----GGNY 59  
Populus_trichocarpa_XP_0023232  MTESRDRLSRAVDIAAIFAARRQSMNLGIYQDRP-ELDMALFGSP---R------------------TN-TAIR-NQTVGV----GTITGRG----RGRL 68  
Populus_trichocarpa_XP_0023309  MPVSRDRLSSPVDIAALFAARRQSRILGVYQDQP-ELDMALFGSP---R------------------PN-AATR-TQTVGA----GTIAVRG----RGGL 68  
Medicago_truncatula_AC141114_1  MPEARDRRVIPLDVDTLF--RRPF---SAVFQESEPLS--VTPAPA---------------------PF-TAGL-----------------------DLF 48  
Ricinus_communis_XP_002532403   MPEARDRLSRPIDIATVFSRRRSG-LIGVYQDQP-DLETALFGSPITSR------------------LD-TATR-TGTVGL-----SPRGRG----RGSF 69  
Vitis_vinifera_XP_002277253     MPESRDRLSRPEDIAELFLRRRSG-ILGILADGS-ERSSNLFASPSR--------------------RE-TTTR-TTTLGARGATGILASRGGGVGRGGF 76  
Oriza_sativa_Os04g39670         MPEMRDSK-RTA------------------------------------------------------------------------LGEL---S---GGGGF 21  
Oriza_sativa_Os02g37850         MPEVRNSGGRAA------------------------------------------------------------------------LADP---S---G-GGF 21  
Sorghum_bicolor_JGI5057365      MPDSRDGR-RAA------------------------------------------------------------------------LADL---SSGVGGGGF 24  
Sorghum_bicolor_JGI4979131      MPQLRTAS-RPV------------------------------------------------------------------------LARN---S---T-GGI 20  
Sorghum_bicolor_JGI5055355      MHESRTAR-RPA------------------------------------------------------------------------LADI---S---G-GGF 20  
Picea_sitchensis_D5AAH2         MPEELH---------VSGLRETLH---LNPSDDG-SSNVDSDQQTQQHINTGTTTYHRFETHQHATNPRNTEMR-TPEA--------------------- 65  

                                        110       120       130       140       150       160       170       180       190       200         
                                ....|....|....|....|....|....|....|....|....|....|....|....|....|....|....|....|....|....|....|....|
Arabidopsis_thaliana_OSD1       TTWRPGN------GRG-GH---T---PFRLPQ-GRENM-P--IVTAR--RGRGG--------GLLPSWYPRTPLRDITHIVRAIERRRGAGTGGDDGRVI 133 
Arabidopsis_thaliana_UVI4       GIRRTGG------GRR-GQ---I---QFRSPQ-GRENM-S--LGVTR--RGRARA-----SNSVLPSWYPRTPLRDISAVVRAIERRR-ARMGEGVGRDI 147 
Arabidopsis_lyrata_XP_00287644  STWRPGN------GRG-GH---S---PFRLSQ-GRENNMP--MVSAR--RGRGP--------SLLPSWYPRTPLRDITHIMRTIERRRGAGIGGDDGRDI 139 
Arabidopsis_lyrata_JGI903574    GIRRTGG------GRR-GQ---I---QFRSPQ-GRENM-S--LGVTR--RGRARA-----SNSVLPSWYPRTPLRDVSAVVRAVERRR-ARMGEGVGRDI 146 
Brassica_rapa_EX107108          GIRRTGG------G-----------------------------GGRR--RGRARA-----SASVLPSWYPRTPLRDVSSVVRAIERRR-ARVG-----DV 110 
Brassica_rapa                   SSWRPGH------GNGSGQ---S---PFRFSQ-GRENV-T--MASAR--RGRGGA-----SGSLLPSWYPRTPLRDITHIMRAIERKRRAGMGVESALGG 136 
Populus_trichocarpa_XP_0023232  GTPR-----------GRGG---------WTPL-DRENMPP--PGSAR--RRRGRG-----SNSLLPSWYPRTPLRDITAVVRAIERR--GRLGGSDGREI 136 
Populus_trichocarpa_XP_0023309  GTPR-----------GRGG---------RTTL-GRENIPP--PGSAR--RGRGRG-----SNSVLPAWYPRTPLRDVTAVVRAIERRR-ERLGGSDGLEI 137 
Medicago_truncatula_AC141114_1  FTERTPV------RRE-VA---R---ARRS-P-GSENTP---PTTAR--RGRGRA---TASRSALPSWYPRTPLQDITAIVRAIERRR-ERQGTEEIEQT 124 
Ricinus_communis_XP_002532403   GTPRNQT------LRGRHP---------YVTI-GRENTPV----TGR--RG--NG-----NRSVLPSWYPRTPLRDITAIVRAIERRR-ELLGEGRAQEI 139 
Vitis_vinifera_XP_002277253     GTPRIGT------GRGRGR---A---VYRSPLFGRENTPA--TGSGR--RGRGRS-----GNSVLPSWYPRTPLRDITHVVRAIERRR-ARLREIDGQQI 154 
Oriza_sativa_Os04g39670         FIRRVASP-GALAARGPGKPLAR---RFIRPSNNKENVP---PVWAV--KATA-----TKRRSPLPDWYPRTPLRDITAIAKAIQRSR-LRIAAAQQRSQ 106 
Oriza_sativa_Os02g37850         FIRRTTSPPGAV----AVKPLAR---RALPPTSNKENVP---PSWAVTVRAT------PKRRSPLPEWYPRSPLRDITSVVKAVERKSRLGNAAVRQQIQ 105 
Sorghum_bicolor_JGI5057365      FIRRVASP-RALAVRGAGKPLAR---RYMSPSRNKENLL---PIWAL--RAT------PAKRSPLPGWYPRTPLRDITAIAKAIQRSR-ARIAAAQQQSQ 108 
Sorghum_bicolor_JGI4979131      FIRRRVASPG-----GAVKPLAR---RVRTHFSNKENVP---PVGAA--RAK------PKRRSPLPDWYPRSPLRDITSIVKALEKRNRLEEDAARQHIQ 101 
Sorghum_bicolor_JGI5055355      FIRRVESP-GAVLVKGAVKPLAR---RALSQSSNKENVP---PVGAV--RGA------PKRKSPLPDWYPRTPLRDITSIVKAIERRSRLQNAATEQTIL 105 
Picea_sitchensis_D5AAH2         --------------------LARALLRARESIQNKENLVPEKPGSQR--RGRRRRLVVSTSRSPLPSDYPRAPLQDITAYMHALQRSR-SRTRTRMQLHG 142 

                                        210       220       230       240       250       260       270       280       290       300         
                                ....|....|....|....|....|....|....|....|....|....|....|....|....|....|....|....|....|....|....|....|
Arabidopsis_thaliana_OSD1       EIP----TH-RQVG-VL--ESPVPLS---GEHKCSMVTPGP----S--VGFKRSCPPSTA--KVQK----------MLLDI-----TK---EIAE--EEA 194 
Arabidopsis_thaliana_UVI4       ETP----TP-QQLG-VL--DSLVPLSGAHLEHDYSMVTPGP----S--IGFKRPWPPSTA--KVHQ----------ILLDI-----TR---ENTGEE--- 210 
Arabidopsis_lyrata_XP_00287644  EIP----TH-QQVG-VL--ESPVPLS---GEHKCSIVTPGP----S--VGFKRSCPPSTA--KVHK----------MLLDI-----TK---EIAE--EEA 200 
Arabidopsis_lyrata_JGI903574    ETP----TP-QQLG-VL--DSLVPLSGAQLEHDYSMVTPGP----S--VGFKRPWPPSTA--KVHQ----------ILLDI-----TR---ENTGEE--- 209 
Brassica_rapa_EX107108          ETP----TP-QQLE-VVLDDSLAPVSG---ERNYSMVTPGP----S--VGFKRPWPPSTA--KVHQ----------ILLDI-----TR---QSSAEE-EE 174 
Brassica_rapa                   ETP----SH-QQVR-FL--ETPVALA--EDEHNCVMVTPAP----A--VGLKRSCPPSTA--KVHK----------MLLDI-----TK---DISDNDEQA 200 
Populus_trichocarpa_XP_0023232  GSP----MP-QGRM-DP--EFSEATPVAHPEPSNRIMSPKP----T--PAFKG-CPSTIG--KVPK----------ILQHI-----TN---QASGDP--- 198 
Populus_trichocarpa_XP_0023309  RSP----MP-QVRM-NH--DSSEATPVAHLEHSNRIMSPKP----T--TAVKG-CSSTIG--KVPK----------ILQHI-----TN---QASGDP--- 199 
Medicago_truncatula_AC141114_1  GTP----VHANQLT-IF--SDPSSFS-AAIGSSSRVHKKSP----K--SCIKL-KTPYGS--KVPK----------IIIDI-----AKLPAAEDGE---S 189 
Ricinus_communis_XP_002532403   ESP----VP-HAYE-VP--DSSEPSAVAHLEHSNSMMSPIP----S--LQVKR-CPPTVG--KVSK----------ILLDI-----TN---KASDDS--- 201 
Vitis_vinifera_XP_002277253     DIP----IP-QDISDVH--DPILPPSSAQLEQDISMISPSP----T--SGMKL-VPKAVG--KVPK----------ILLDI-----TD---QTGGGS--- 217 
Oriza_sativa_Os04g39670         TPE--QNTP-HCT------EVR--------DSLDVEP-----GINS--TQIVAT----------------------PASS-LAKDSLK-IFSSP---SET 155 
Oriza_sativa_Os02g37850         LSE--DS-S-RSV------DPATPV---QKEEGVPQSTPTPPTQKA--LDAAAPCPGSTQ--AVAS----------TSTAYLAEGKPKASSSSP---SDC 175 
Sorghum_bicolor_JGI5057365      RIE--QS-P-QSV------NVTTPA---QAEQDAPHI-----A-EA--SHAVASGSGSTERETVAN----------PATV-LADDNLN-VSSSP---AES 172 
Sorghum_bicolor_JGI4979131      WNE--DS-P-QPV------DPTTTV---HAEHSDPDSQSTQT-QET--LGVV-ASPGSTS--AVAN----------NVTS-VAEDKQE-ASSSP---SDC 167 
Sorghum_bicolor_JGI5055355      WTE--DS-S-QSV------DPITPA---SAEQGVPTIEGGQ----A--V---------------AR----------HATS-LGDGKLKT-SSSP---FDC 156 
Picea_sitchensis_D5AAH2         ITPPRQPIT-HDSP-VQ--ERSAQEGPLQVEQTTTTIIPSP----VPEQEIEGAVTREET--DVHNLGPERPLMYQTRVDM-----DN---SSTVDT--- 221 

                                        310       320       330       340       350       360       370            
                                ....|....|....|....|....|....|....|....|....|....|....|....|....|....|....|...
Arabidopsis_thaliana_OSD1       GF-ITP-------------EKKLLNSI--------DKVEKIVMAEIQKLK---STPQAKREER---EKRV-RTLMTMR 243 
Arabidopsis_thaliana_UVI4       DA-LTP-------------EKKLLNSI--------DKVEKVVMEEIQKMK---STPSAKRAER---EKRV-RTLMSMR 259 
Arabidopsis_lyrata_XP_00287644  GF-ITP-------------EKKLLNSI--------DKVEKIVMAEIQKLK---STPHAKREER---EKRV-RTLMSMR 249 
Arabidopsis_lyrata_JGI903574    DA-LTP-------------QKKLLNSI--------DKVEKVVMEEIQKMK---STPSAKRAER---EKRV-RTLMSMR 258 
Brassica_rapa_EX107108          EA-LTP-------------QKKLLNSI--------DKVEKVVMEEIQKMK---STPSAKRAER---EKRV-RTLMSMR 223 
Brassica_rapa                   RF-ITP-------------EKKLLNSI--------DVVEKIVMAEIQKLK---STPLAKRQER---EKRV-KTLMSMR 249 
Populus_trichocarpa_XP_0023232  EC-LTP-------------QKKLLNSI--------DTVEKVVMEELQKLK---RTPSAKKAER---EKRV-RTLMSMR 247 
Populus_trichocarpa_XP_0023309  DS-LTP-------------QKKLLNSI--------DTVEKVVMEELRKMK---RTPSARKAER---EKRV-RTLMSMR 248 
Medicago_truncatula_AC141114_1  EL-LTP-------------QKKLLHSI--------DIIEREVKQELMKLK---RTPTAKKAEH---QKRV-RTLMSMR 238 
Ricinus_communis_XP_002532403   EF-LTP-------------QKKLLNSI--------DTVEKEVMEELRKLK---RTASAKKAER---EKKV-RTLMSLR 250 
Vitis_vinifera_XP_002277253     DF-LTP-------------QKKLLNSI--------DTVEKAVMDELGKLK---RTPSAKRAEQ---EKRV-RTLMSMR 266 
Oriza_sativa_Os04g39670         SL-VTPSKPMDPVLLDD-MEKKLSSSI--------EQIEKMVRRNL---K---RTPKAAAAQPSKRAIQR-RTLMSMR 216 
Oriza_sativa_Os02g37850         SF-QTPSRPNDPALADL-MEKELSSSI--------EQIEKMVRKNL---K---RAPKAAQPSKVTIQK---RTLLSMR 234 
Sorghum_bicolor_JGI5057365      SL-NTPSKPMDPALADI-VEKKLSSSI--------EKIEKLVRKNM---K---RTPKAARASRRA--TQR-RNLMSMR 231 
Sorghum_bicolor_JGI4979131      LQ-MAPSKPNDPSPAD--LEKKMSSSI--------EQIEKMVRRHM---K---ETPKAAQPSKLVV--QR-RILMSMR 225 
Sorghum_bicolor_JGI5055355      SLQATPSKPNDPALADL-MEKKLSNSI--------EQIEKMVRRNL---K---KTPKAAQP--SKRTIQS-RILMSMR 216 
Picea_sitchensis_D5AAH2         ----RPGLQQDNQVQEISPRDDSRLTERTFGRAFGTVLQSGPQNEISGRDRNGSCPQPKKTSS---SKNTAKTLMKMR 292 
