# Supplementary figures and images for: OSD1 Promotes Meiotic Progression via APC/C Inhibition and Forms a Regulatory Network with TDM and CYCA1;2/TAM
Source: PLoS Genet. 2012 Jul 26;8(7):e1002865. doi: 10.1371/journal.pgen.1002865 (PMC3406007; doi:10.1371/journal.pgen.1002865)

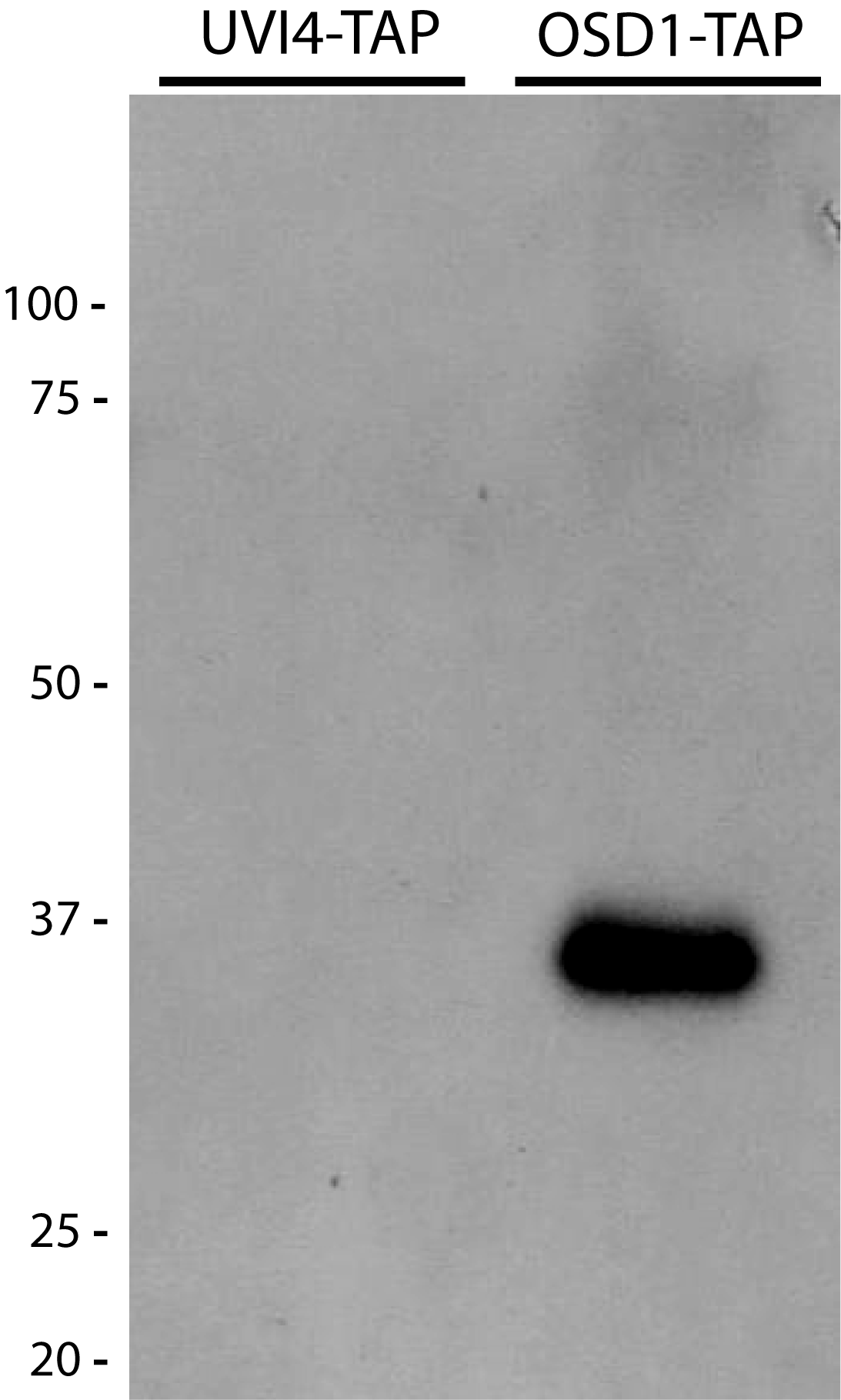

Supplement: Figure S2 — The anti-OSD1 antibody is specific. Probing of UVI4 and OSD1 TAP elutions with anti-OSD1 antibody shows it recognizes specifically OSD1. (TIF) [file pgen.1002865.s002.tif]

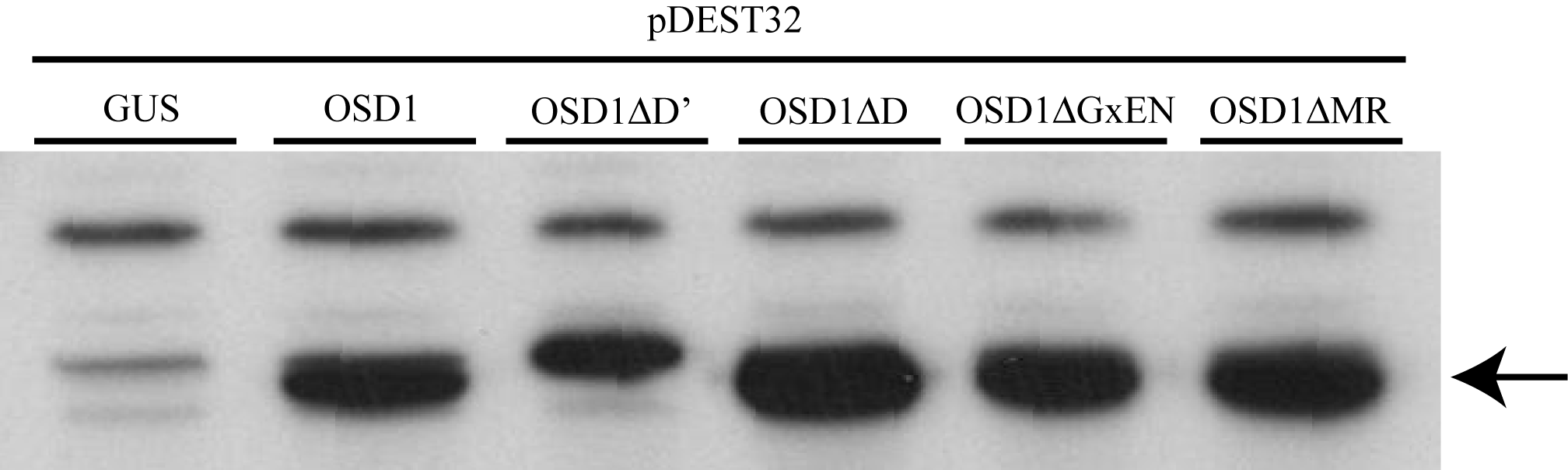

Supplement: Figure S3 — Mutated version of OSD1 are stably expressed in yeast. Protein extracts [47] of yeasts transformed with wild type or mutated version of OSD1 were probed with anti-OSD1 antibody. Wild type or mutated version of OSD1 showed similar expression levels. (TIF) [file pgen.1002865.s003.tif]

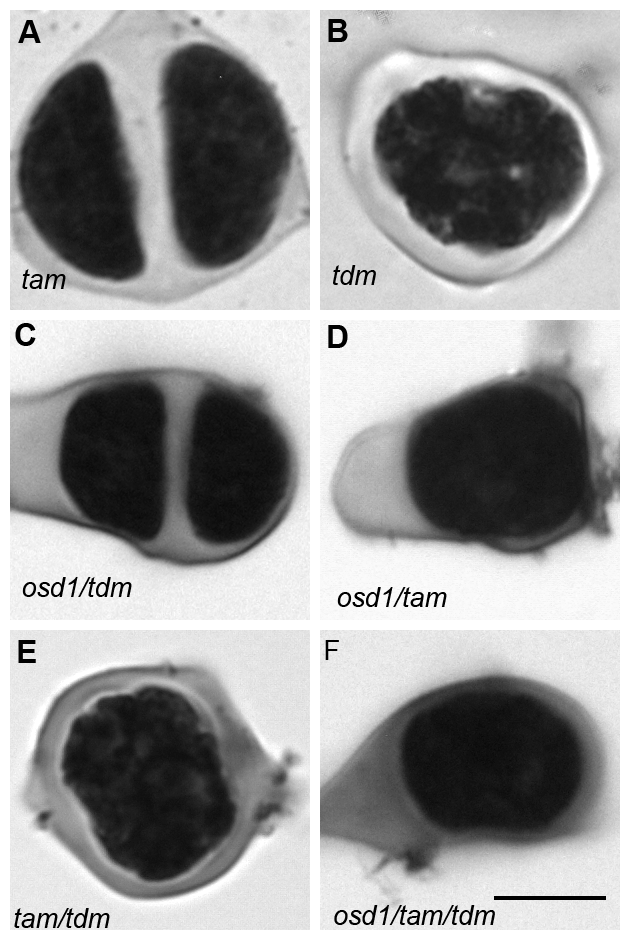

Supplement: Figure S4 — Meiotic product stained by toluidine blue. (A) tam-2. (B) tdm-3. (C) osd1-3/tdm-3. (D) osd1-3/tam-2. (E) tam-2/tdm-3. (F) osd1-3/tam-2/tdm-3. Scale bar = 10 µM. (TIF) [file pgen.1002865.s004.tif]

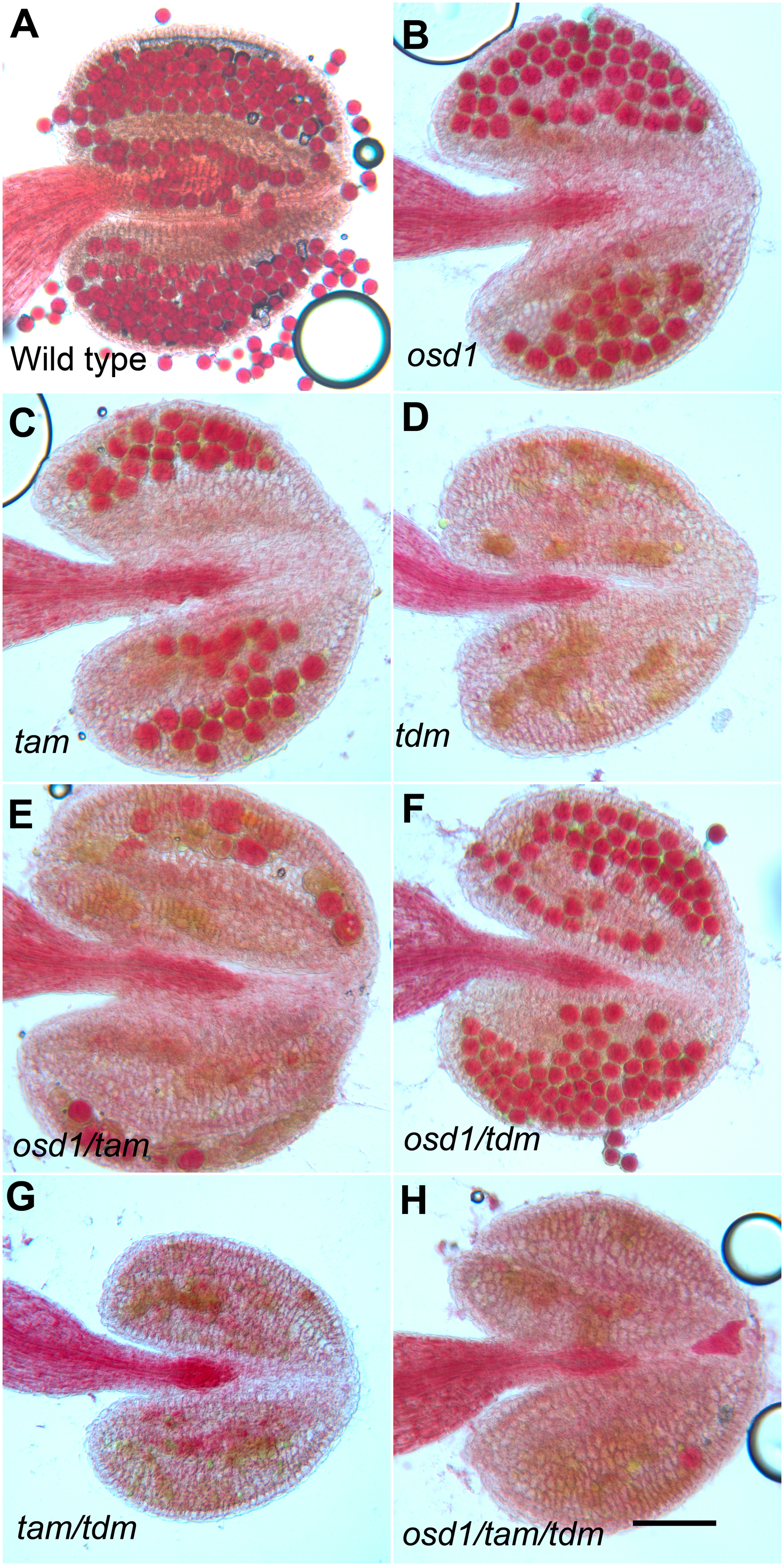

Supplement: Figure S5 — Alexander staining of anthers. Viable pollen grains are stained in red. (A) Wild type. (B) osd1-3 (C) tam-2. (D) tdm-3. (E) osd1-3/tam-2. (F) osd1-3/tdm-3. (G) tam-2/tdm-3. (H) osd1-3/tam-2/tdm-3. Scale bar = 100 µM. (TIF) [file pgen.1002865.s005.tif]

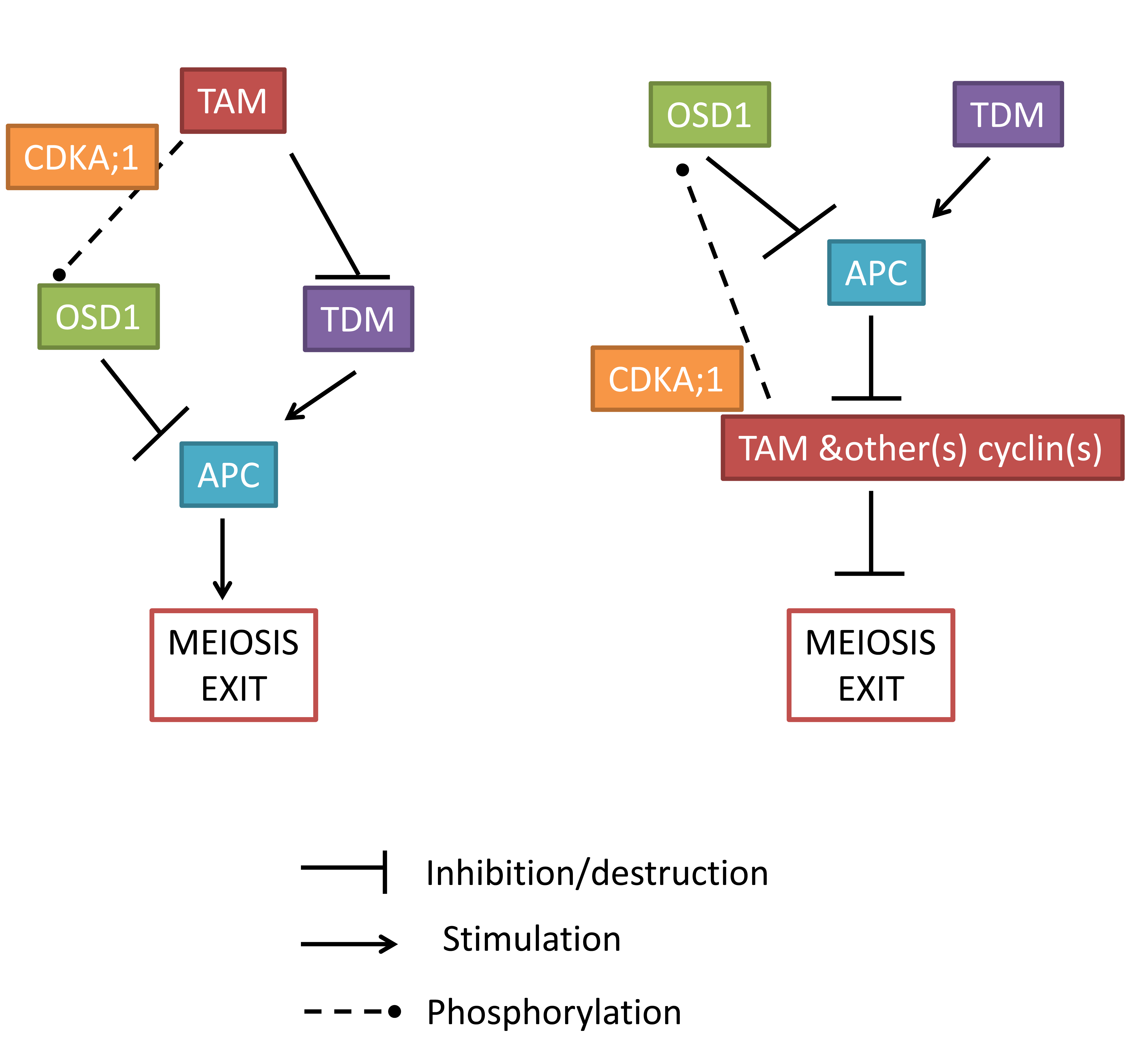

Supplement: Figure S6 — Two alternative models for the OSD1, CYCA1;2/TAM and TDM functional network. (TIF) [file pgen.1002865.s006.tif]
