# Supplementary material for: OSD1 Promotes Meiotic Progression via APC/C Inhibition and Forms a Regulatory Network with TDM and CYCA1;2/TAM
Source: PLoS Genet. 2012 Jul 26;8(7):e1002865. doi: 10.1371/journal.pgen.1002865 (PMC3406007; doi:10.1371/journal.pgen.1002865)
Supplement: Table S1 — Genetic analysis of osd1/uvi4 transmission. (PDF) [file pgen.1002865.s007.pdf]

	Parental genoptype \ offspring genotype	*osd -/-* *uvi4 -/-*	*osd +/+* *uvi4 +/+*	*osd +/-* *uvi4 +/+*	*osd -/-* *uvi4 +/+*	*osd -/-* *uvi4 +/-*	*osd +/+* *uvi4 +/-*	*osd +/-* *uvi4 +/-*	*osd +/+* *uvi4 -/-*	*osd +/-* *uvi4 -/-*
Selfing	**Expected**	**6,25%**	**6,25%**	**12,50%**	**6,25%**	**12,50%**	**12,50%**	**25,00%**	**6,25%**	**12,50%**
	observed *osd1-1 +/- uvi4 +/-* (n=169)	0,00%	6,50%	28,40%	4,14%	4,73%	15,90%	24,85%	10,65%	4,73%
	observed *osd1-2 +/- uvi4 +/-* (n=224)	0,00%	7,14%	13,83%	8,49%	5,80%	23,21%	22,76%	4,91%	13,83%
	**Expected**	**25,00%**							**25,00%**	**50,00%**
	observed *osd1-2 +/- uvi4 -/-* (n=272)	0,00%							45,00%	55,00%
Backcrosses	**Expected**		**25,00%**	**25,00%**			**25,00%**	**25,00%**		
	observed WT♀ x *osd1-2 +/- uvi4 +/-* ♂ (n=76)		30,30%	19,70%			30,30%	19,70%		
	observed *osd1-2 +/- uvi4 +/-* ♀ x WT ♂ (n=53)		35,80%	30,20%			32,10%	1,90%		
	**Expected**						**50,00%**	**50,00%**		
	observed WT♀ x *osd1-2 +/- uvi4 -/-* ♂ (n=68)						55,90%	44,10%		
	observed *osd1-2 +/- uvi4 -/-* ♀ x WT ♂ (n=44)						91,00%	9,00%		
